# Timing‐Dependent Clearance of p16‐Positive Cells Mitigates Radiation‐Induced Accelerated Aging

**DOI:** 10.1111/acel.70693

**Published:** 2026-08-26

**Authors:** Karla Valdivieso, Melanie Weigand, Daniela G. Costa, Gung Lee, Nick Pirius, Helene Martini, Shivangi Oberai, Christina Inman, Yi Zhu, Thomas von Zglinicki, Sundeep Khosla, Nathan LeBrasseur, João F. Passos, Tamara Tchkonia, James L. Kirkland, Diana Jurk

**Affiliations:** ^1^ Department of Physiology and Biomedical Engineering Mayo Clinic Rochester Minnesota USA; ^2^ Robert and Arlene Kogod Center on Aging Mayo Clinic Rochester Minnesota USA; ^3^ Faculty of Pharmacy University of Coimbra Coimbra Portugal; ^4^ Center for Neuroscience and Cell Biology (CNC‐UC) and Center for Innovation in Biomedicine and Biotechnology (CIBB) University of Coimbra Coimbra Portugal; ^5^ Molecular Pharmacology and Experimental Therapeutics Graduate Program Mayo Clinic Graduate School of Biomedical Sciences Rochester Minnesota USA; ^6^ Department of Cellular and Integrative Physiology UT Health San Antonio San Antonio Texas USA; ^7^ The Sam and Ann Barshop Institute for Longevity and Aging Studies UT Health San Antonio San Antonio Texas USA; ^8^ Biosciences Institute, Faculty of Medical Sciences Newcastle University Newcastle upon Tyne UK; ^9^ Center for Advanced Gerotherapeutics, Department of Medicine Cedars‐Sinai Health Sciences University Los Angeles California USA; ^10^ Department of Neurology Mayo Clinic Rochester Minnesota USA

**Keywords:** aging, BBB, brain, cognition, irradiation, senescence, senolytic, therapy induced senescence

## Abstract

Genotoxic stress induced by cancer therapies is increasingly recognized as a driver of accelerated aging in long‐term cancer survivors, yet the mechanisms responsible for the emergence of age‐related dysfunction months to years after treatment remain poorly understood. Here, we use sublethal whole‐body irradiation as a model of systemic genotoxic stress to test whether senescent cells contribute to the progression of post‐therapy age‐related dysfunction and whether the benefits of senescent cell clearance depend on the timing of intervention. Using the *INK‐ATTAC* mouse model, we selectively eliminated p16^Ink4a^‐positive cells either early (1 month) or later (4 months) after irradiation. Early clearance had no effect on lifespan or functional outcomes. In contrast, delayed clearance markedly reduced frailty, improved neuromuscular and cognitive function, restored blood–brain barrier integrity, improved hepatic metabolic dysfunction, and increased median survival, with the survival benefit being most evident in female mice. Mechanistically, irradiation induced an early p21^Cip1^‐associated stress response and later accumulation of p16^Ink4a^‐positive cells in the brain and liver, which was associated with inflammation and tissue dysfunction. Clearance of p16^Ink4a^‐positive cells at the later stage attenuated these changes. Together, these findings identify p16^Ink4a^‐positive cells as key drivers of the radiation‐induced accelerated aging‐like state that emerges progressively after genotoxic stress. They also show that the efficacy of senescence‐targeted interventions depends on when treatment is initiated, with implications for improving long‐term outcomes in cancer survivors.

## Graphical abstract

Genotoxic stress induces a progressive accelerated aging‐like phenotype driven by the delayed accumulation of p16^Ink4a^‐positive senescent cells. Selective clearance of these cells improves healthspan, tissue function, and survival only when initiated after senescent cells have accumulated, highlighting a critical therapeutic window for senescence‐targeted interventions.

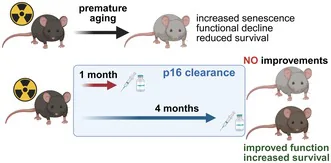

## 1 Introduction

Genotoxic stress is a central component of many cancer therapies. Chemotherapy and radiation therapy are widely used to induce senescence or cell death in malignant cells, thereby limiting tumor progression (Prasanna et al. 2021; Sabin and Anderson 2011). As survival after cancer treatment has improved, it has become increasingly clear that exposure to non‐lethal doses of radiation leading to genotoxicity has long‐term consequences. Cancer survivors can display features of an accelerated organismal aging‐like state, including frailty, cognitive decline, and reduced lifespan (Ness et al. 2013; Krull et al. 2018; Armstrong et al. 2014). Notably, these phenotypes may emerge long after treatment has ended, suggesting that the early genotoxic injury initiates mechanisms that accelerate age‐related processes over time, rather than causing immediate tissue failure (Demaria et al. 2017). However, the cellular mechanisms that convert acute damage into progressive age‐related dysfunction, and the time window during which they become functionally relevant, remain poorly defined.

Senescent cells have emerged as key contributors to aging and age‐related disease (Baker et al. 2016; Ogrodnik et al. 2017; Ogrodnik et al. 2019). While senescence can play beneficial roles in tumor suppression and tissue repair, the persistence of senescent cells is associated with chronic secretion of SASP factors that promote inflammation, alter tissue microenvironments, and induce senescence in neighboring and distant cells (Nelson et al. 2012; Rodier et al. 2009; Xu et al. 2018). Through these non‐cell‐autonomous effects, senescent cells can amplify, spread, and induce tissue dysfunction over time, suggesting that their pathological impact may depend on both their abundance and persistence (Baker et al. 2011).

Recent studies have highlighted that p16^Ink4a^ and p21^Cip1^‐expressing cells represent distinct senescent populations with different tissue distributions, transcriptional programs, and biological functions. Although both contribute to age‐related pathology, accumulating evidence suggests that these populations exhibit different temporal dynamics following tissue injury and may respond differently to senescence‐targeting interventions (Fielder et al. 2022).

Based on these observations, we hypothesize that sublethal genotoxic stress accelerates aging processes through the gradual accumulation of senescent cells, and that clearance of these cells decelerates aging processes and confers long‐term functional benefits. We further hypothesize that the efficacy of senescence‐targeted interventions depends on timing, such that clearance is beneficial only once senescent cells have accumulated to levels that meaningfully contribute to tissue dysfunction (Passos et al. 2007; Sharpless 2003). Our overarching goal is to determine whether a time‐dependent approach to senescent cell clearance can improve long‐term outcomes after radiation exposure, with relevance to cancer therapy and survivorship.

To test our hypothesis, we used sublethal whole‐body irradiation as a model of systemic genotoxic stress and accelerated aging. While this approach does not fully recapitulate clinical radiotherapy, it provides a controlled proof‐of‐concept system to interrogate the temporal relationship between genotoxic stress, senescent cell accumulation, and organismal aging. Using the *INK‐ATTAC* mouse model, we selectively eliminated p16^Ink4a^‐expressing cells at two distinct time points: an early intervention 1 month after irradiation and a late intervention 4 months after irradiation.

By comparing survival, frailty, and cognitive function across these intervention windows, we directly assessed whether the benefits of senescent cell clearance depend on the timing of senescent cell emergence. We found that early clearance of p16^Ink4a^‐positive cells did not improve functional outcomes. In contrast, late clearance markedly reduced frailty, improved cognitive performance, restored blood–brain barrier integrity, and extended survival. Consistent with these findings, analysis of p16^Ink4a^ mRNA expression revealed a delayed increase following irradiation in key tissues, including the liver and brain, indicating that p16^Ink4a^ senescent cells become prominent only at later stages after genotoxic stress.

Together, these results suggest that p16 positive cells act as late‐emerging drivers of the radiation‐induced accelerated aging‐like state and highlight the importance of timing interventions targeting p16^Ink4a^ positive cells for the success of senescence‐targeted therapies.

## 2 Results

### 2.1 Whole‐Body Irradiation Induces Long‐Term Functional Decline and Cognitive Impairment

**FIGURE 1:**
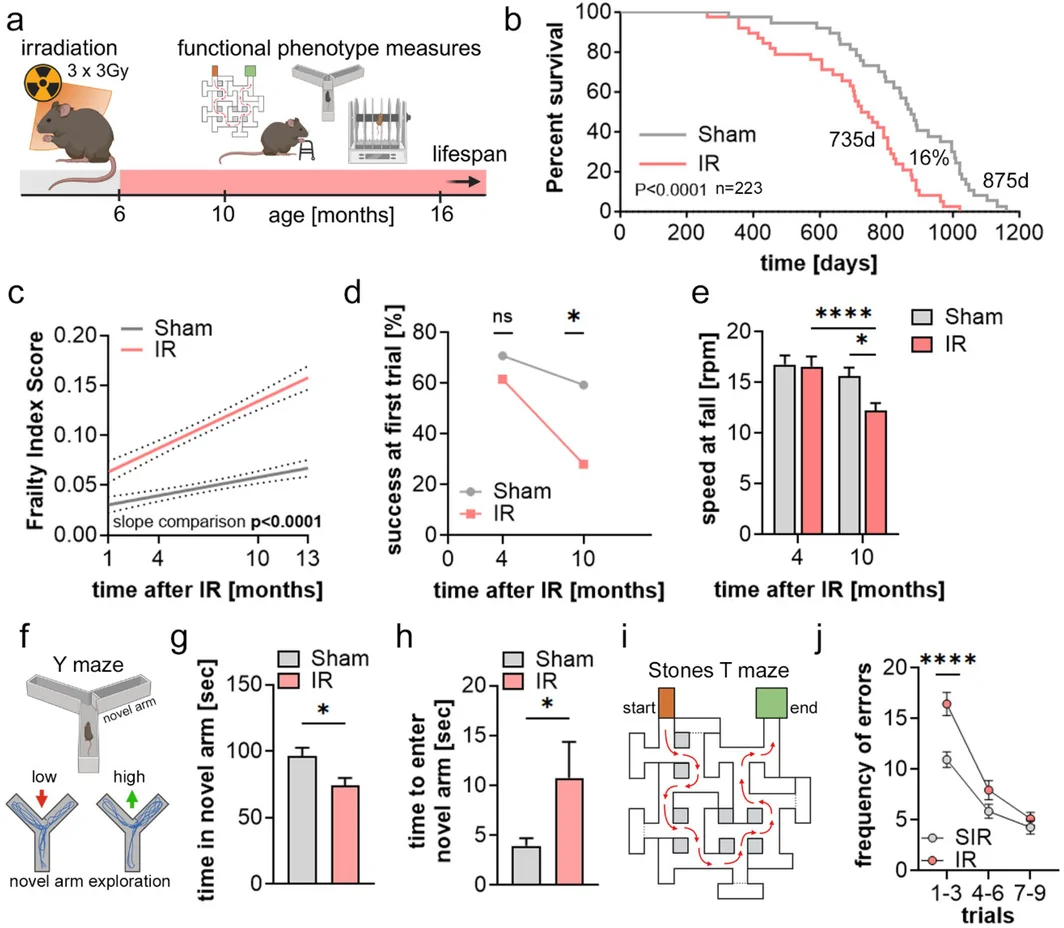
Whole‐body irradiation induces long‐term functional decline and cognitive impairment. (a) Experimental design showing fractionated whole‐body irradiation (3 × 3 Gy) at 6 months of age followed by longitudinal assessment of functional phenotypes and survival. (b) Kaplan–Meier survival curves of sham (Sham) and irradiated (IR) mice. IR mice exhibited significantly reduced survival compared to Sham (*p* < 0.0001). Median survival: Sham 875 days, IR 735 days; median lifespan extension in % are shown. (c) Frailty Index Scores (FIS) over time following irradiation, with linear regression lines indicating the rate of frailty progression. IR mice showed accelerated frailty progression compared to Sham (comparison of slopes, *p* < 0.0001). (d) Wire hanging test showing the percentage of mice successful in the first trial at different time points after irradiation. No statistically significant difference was observed at 4 months (*p* = 0.3038), whereas performance was significantly reduced in IR mice at 10 months (*p* = 0.0226). (e) RotaRod performance shown as speed at fall (rpm). A significant interaction between time and irradiation was observed (*p* = 0.0023), with IR mice showing reduced performance at 10 months (*p* = 0.0128) but not at 4 months (*p* = 0.8719). (f) Y‐maze test assessing novel arm exploration; schematic illustration of the task is shown. (g) Time spent in the novel arm during the Y‐maze. IR mice (10 months after IR) spent less time in the novel arm compared to Sham (*p* = 0.0157). (h) Latency to enter the novel arm in the Y‐maze. IR mice exhibited increased latency compared to Sham (*p* = 0.0268). (i) Schematic illustration of the Stone T‐maze task. (j) Stone T‐maze performance shown as frequency of errors across trial blocks (trials 1–3, 4–6, 7–9). A significant interaction between trial block and irradiation was observed (*p* = 0.0164). IR mice (10 months after IR) made more errors overall (main effect *p* < 0.0001), with significant differences during trials 1–3 (*p* < 0.0001). Statistical analyses: Survival curves were analyzed by log‐rank (Mantel‐Cox) test. Frailty progression was assessed by linear regression with comparison of slopes. Wire hanging performance was analyzed by Fisher's exact test. RotaRod performance was analyzed by mixed‐effects model. Y‐maze data were analyzed by unpaired two‐tailed *t*‐test or Mann–Whitney test as appropriate. Stone T‐maze performance was analyzed by two‐way ANOVA with Šídák's multiple comparisons test. Sample sizes: (b) Sham *n* = 37, IR *n* = 38. (c) Sham *n* = 34, IR *n* = 33. (d) 4 m: Sham *n* = 77, IR *n* = 73, 10 m: Sham = 32, IR = 32. (e) 4 m: Sham *n* = 33, IR *n* = 29, 10 m: Sham = 32, IR = 29. (g, h) Sham *n* = 24, IR *n* = 21. (j) Sham *n* = 22, IR *n* = 22. Data are presented as mean ± SEM. *p < 0.05, **p < 0.01, ***p < 0.001, ****p < 0.0001.

Genotoxic stress is a well‐established accelerator of biological aging processes, and whole‐body irradiation provides a controlled in vivo model to study this process. To determine the long‐term consequences of systemic genotoxic stress, 6–8‐month‐old mice were exposed to fractionated whole‐body irradiation (3 × 3 Gy) and followed longitudinally for functional outcomes and survival (Figure 1a).

Irradiation significantly reduced survival compared with age‐matched sham‐treated controls (Figure 1b). Median survival decreased from 875 days in sham mice to 735 days following irradiation, representing a 16% reduction in median survival. Sex‐stratified analyses demonstrated that this effect was predominantly driven by females, in which median survival decreased from 884 to 814 days, whereas only a minimal reduction was observed in males (865 vs. 696.5 days; Figure S1a,b).

To assess biological aging, we quantified frailty using a 30‐parameter clinical frailty index (Whitehead et al. 2014). Irradiated mice exhibited accelerated frailty progression over time relative to sham controls, as reflected by a significantly steeper slope of frailty accumulation (Figure 1c). Sex‐stratified analyses confirmed progressive age‐associated increases in frailty in both male and female cohorts (Figure S1c,d), consistent with sustained functional decline following irradiation.

Motor performance declined progressively after irradiation. In the wire hanging test, no differences were observed at early time points; however, irradiated mice displayed reduced success rates at later time points (Figure 1d). When analyzed separately by sex, this late impairment was evident in males, whereas females did not show significant differences (Figure S1c–f).

Similarly, RotaRod testing indicated that irradiation accelerated the age‐related decline in motor performance (Figure 1e). Reduced performance was observed at later timepoints following irradiation. Sex‐stratified analyses demonstrated that this effect was primarily evident in males, while females did not show significant impairment (Figure S1g,h).

Beyond physical decline, irradiation impaired spatial learning and memory. In the Y‐maze, irradiated mice exhibited reduced exploration of the novel arm and increased latency to enter it, consistent with impaired spatial recognition memory (Figure 1f–h). Sex‐stratified analyses revealed that reduced novel arm exploration was significant in males, whereas females showed no significant differences (Figure S1i–l).

To further assess learning and executive function, mice were tested in the Stone T‐maze. Although both sham and irradiated mice improved across trial blocks, indicating learning, irradiated mice made more errors during early trials (Figure 1j). Sex‐stratified analyses confirmed increased early trial errors in both males and females (Figure S1m,n). Irradiated mice also required more time to complete the maze during early trial blocks, an effect that was most evident in males and remained detectable in pooled analyses (Figure S1o–q).

Collectively, these findings show that systemic genotoxic stress drives a durable and progressive decline across survival, physical function, and cognition, reflecting key features of accelerated biological aging and establishing a rigorous framework for testing whether senescent cell clearance can delay the onset of irradiation‐induced age‐related phenotypes.

### 2.2 Early Intervention Targeting p16^Ink4a^ ‐Positive Senescent Cells Fails to Alleviate Irradiation‐Induced Functional Decline

**FIGURE 2:**
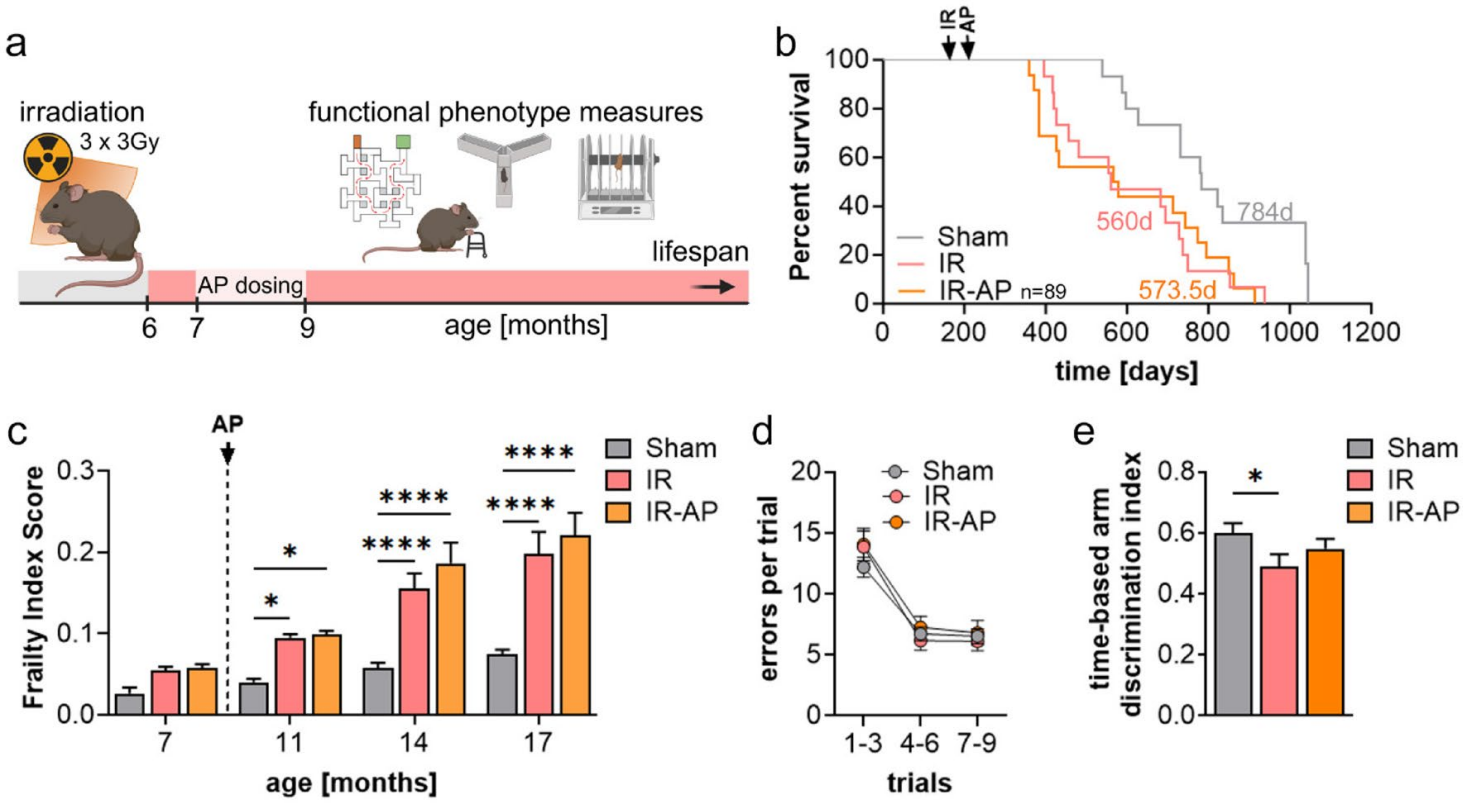
Early senolytic intervention fails to attenuate irradiation‐induced functional decline. (a) Experimental design illustrating early AP treatment initiated 1 month (1 m) after irradiation (3 × 3 Gy) with longitudinal assessment of functional phenotypes and survival. (b) Kaplan–Meier survival curves of sham (Sham), irradiated (IR), and irradiated mice treated with AP (IR‐AP) following early intervention. Survival analysis was performed in females only. Sham vs. IR, *p* = 0.0042; IR vs. IR‐AP, *p* = 0.9874. Median survival: Sham 784 days, IR 560 days, and IR‐AP 573.5 days. (c) Frailty index scores over time following early AP treatment. IR mice exhibited increased frailty compared to Sham at 11–17 months (*p* < 0.05–0.0001), whereas IR‐AP did not significantly differ from IR at any timepoint. (d) Stone T‐maze performance shown as number of errors across trial blocks (1–3, 4–6, 7–9). No significant differences were observed between groups 10 months after IR. (e) Y‐maze time‐based arm discrimination index following early treatment. IR vs. Sham, *p* = 0.0478; IR vs. IR‐AP, *p* = 0.4113 at 10 months after IR. Statistical analyses: Survival curves were analyzed by log‐rank (Mantel‐Cox) test. Frailty and behavioral outcomes were analyzed by ordinary or two‐way ANOVA as indicated, followed by Tukey's or Dunnett's multiple comparisons tests. Sample sizes: (b) Sham *n* = 15, IR *n* = 15, IR‐AP *n* = 16 (females only). (c) Sham *n* = 32–30, IR *n* = 30–28, IR‐AP *n* = 32–30 across timepoints. (d) Sham *n* = 28, IR *n* = 20, IR‐AP *n* = 20. (e) Sham *n* = 26, IR *n* = 20, IR‐AP *n* = 21. Data are presented as mean ± SEM.*p < 0.05, **p < 0.01, ***p < 0.001, ****p < 0.0001.

To determine whether elimination of p16^Ink4a^‐positive senescent cells can mitigate irradiation‐induced premature aging, we induced senescent cell clearance 1 month after whole‐body irradiation using the *INK‐ATTAC* mouse model (Figure 2a). In this model, Caspase‐8 is expressed under control of the p16^Ink4a^ promoter and is activated by administration of the dimerizer AP20187 (AP), enabling selective ablation of p16^Ink4a^‐expressing cells.

Early clearance of p16^Ink4a^‐positive cells did not improve survival after irradiation. Kaplan–Meier analysis showed that irradiated mice had reduced survival compared with sham controls (median survival: 560 vs. 784 days, respectively). AP Treatment 1 month after irradiation did not significantly extend survival, with treated mice exhibiting a median survival of 573.5 days, similar to untreated irradiated mice (Figure 2b).

Early senescent cell clearance also failed to slow the progression of frailty. Irradiated mice developed progressively higher frailty scores compared with sham controls, and AP treatment had no effect on this trajectory (Figure 2c). Similar results were observed in both male and female mice, with no difference in the rate of frailty progression between AP‐treated and untreated irradiated animals (Figure S2a,b).

RotaRod testing revealed no improvement in AP‐treated irradiated mice compared with irradiated controls in either sex (Figure S2c,d), indicating that p16^Ink4a^‐positive senescent cell clearance did not prevent irradiation‐associated motor decline.

Cognitive function was similarly unaffected by early senescent cell clearance. In the Stone T‐maze, AP‐treated irradiated animals made a similar number of errors as untreated irradiated mice across trial blocks (Figure 2d). In the Y‐maze, irradiated mice displayed impaired time‐based arm discrimination relative to sham controls; this deficit was not rescued by AP treatment (Figure 2e). Sex‐stratified analyses further indicated that AP treatment did not significantly improve Y‐maze performance in either males or females (Figure S2e,f).

Together, these findings indicate that elimination of p16^Ink4a^‐positive cells at this stage following irradiation is insufficient to prevent declines in survival, frailty progression, motor coordination, or spatial memory.

### 2.3 Irradiation Induces Progressive Senescence, Persistent DNA Damage, and Neuroinflammatory Changes in the Brain

**FIGURE 3:**
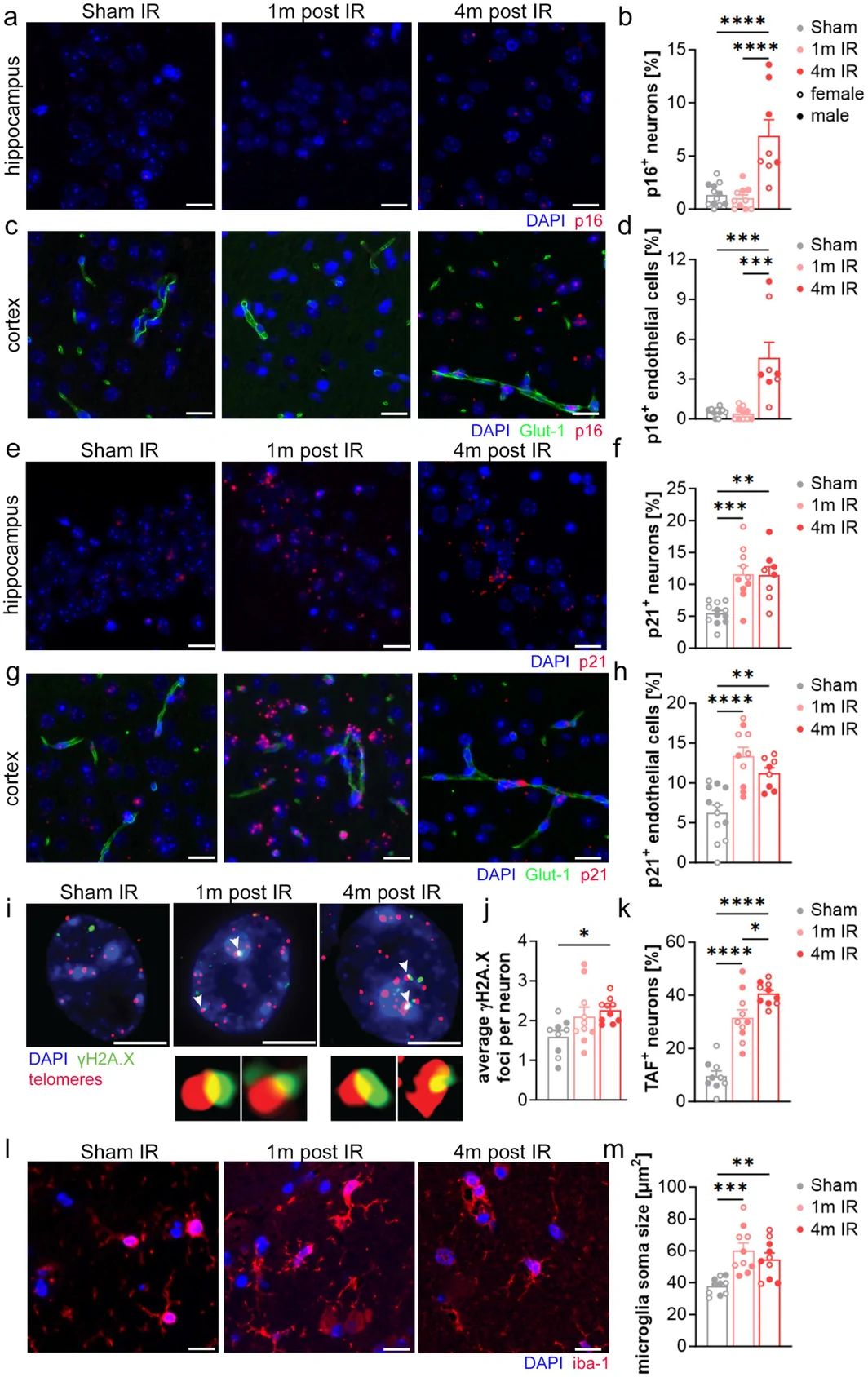
Irradiation induces time‐dependent senescent cell markers and inflammatory changes in the brain. (a) Representative images of RNA‐ISH in hippocampal neurons stained for p16 (red) and DAPI (blue) in Sham, 1 month, and 4 months after irradiation. (b) Quantification of p16^Ink4a^‐positive neurons in the hippocampus. p16^Ink4a^ positivity was significantly increased at 4 months compared to Sham and 1 month post IR (both *p* < 0.0001). (c) Representative images of RNA‐ISH in cortical endothelial cells stained for p16 (red), Glut‐1 (green), and DAPI (blue) in Sham, 1 month, and 4 months after irradiation. Endothelial p16^Ink4a^ positivity was significantly increased at 4 months compared to Sham (*p* = 0.0002) and 1 month post IR (*p* = 0.0001). (d) Quantification of p16^Ink4a^‐positive endothelial cells in the cortex. (e) Representative images of RNA‐ISH in hippocampal neurons stained for p21^Cip1^ (red) and DAPI (blue) in Sham, 1 month, and 4 months after irradiation. (f) Quantification of p21^Cip1^‐positive neurons in the hippocampus. p21^Cip1^ positivity was significantly elevated at 1 month (*p* = 0.0005) and 4 months (*p* = 0.0013) compared to Sham. (g) Representative images of RNA‐ISH in cortical endothelial cells stained for p21^Cip1^ (red), Glut‐1 (green), and DAPI (blue) in sham, 1 month, and 4 months after irradiation. (h) Quantification of p21^Cip1^‐positive endothelial cells in the cortex. p21^Cip1^ positivity was significantly increased at 1 month (*p* < 0.0001) and 4 months (*p* = 0.0051) compared to Sham. (i) Representative images of telomere‐associated DNA damage foci (TAF) in hippocampal neurons stained for telomeres (red), γH2A.X (green), and DAPI (blue) in sham, 1 month, and 4 months after irradiation. TAF were defined by colocalization of telomere and γH2A.X signals (yellow). White arrows indicate TAF. Higher magnification images of TAF are shown below the main panels. (j) Quantification of γH2A.X in neurons. Average γH2A.X‐foci per neuron was significantly increased at 4 months (*p* = 0.0296). (k) Quantification of TAF‐positive neurons. TAF‐positive neurons were significantly increased at 1 month (*p* < 0.0001) and 4 months (*p* < 0.0001) after IR compared to Sham and further increased at 4 months compared to 1 month (*p* = 0.0177). (l) Representative images of hippocampal microglia stained for Iba1 (red) and DAPI (blue). (m) Quantification of microglial soma size. Soma size was significantly increased at 1 month (*p* = 0.0003) and 4 months (*p* = 0.0059) after IR compared to Sham. Scale bars: 20 μm in a, c, e, g, k and 5 μm in i. Open circles represent females and closed circles represent males. Statistical analyses: Ordinary one‐way ANOVA for all panels. Sample sizes: Sham *n* = 10–12; 1 month IR: *n* = 10; 4 months IR: *n* = 8–10 animals per group. Data are presented as mean ± SEM. *p < 0.05, **p < 0.01, ***p < 0.001, ****p < 0.0001.

The absence of functional benefits following early p16^Ink4a^‐positive cell clearance prompted us to examine the temporal dynamics of senescence induction after irradiation. We therefore assessed markers of cellular senescence and inflammation in the brain at 1 and 4 months following whole‐body irradiation (Figure 3a).

RNA‐ISH showed no significant increase in p16^Ink4a^‐positive hippocampal neurons 1 month after irradiation, whereas a robust increase was evident by 4 months post‐irradiation (Figure 3a,b). A similar temporal pattern was observed in cortical endothelial cells, where p16^Ink4a^ positivity was significantly increased at 4 months but not at 1 month (Figure 3c,d). These findings demonstrate that p16^Ink4a^‐driven senescence emerges progressively and becomes prominent only several months after genotoxic stress.

In contrast, p21^Cip1^ expression exhibited an earlier response. Both hippocampal neurons and cortical endothelial cells showed increased p21^Cip1^ positivity at 1‐month post‐irradiation, which remained elevated at 4 months (Figure 3e–h), preceding the accumulation of p16^Ink4a^.

Consistent with persistent DDR signaling, γH2A.X foci and telomere‐associated DNA damage foci (TAF) were significantly increased in hippocampal neurons following irradiation. The average number of γH2A.X foci per neuron was significantly increased at 4 months post‐irradiation (Figure 3j), while TAF‐positive neurons were elevated at both 1 and 4 months, with a further increase observed at 4 months (Figure 3k). These findings indicate sustained DNA damage response and telomere dysfunction following irradiation.

Irradiation was also associated with neuroinflammatory changes. Microglial soma size was increased at both 1 and 4 months (Figure 3l,m), indicative of activation. Microglial number transiently increased at 1 month (Figure S3a,b). Cytokine profiling revealed a robust inflammatory response at 1 month, characterized by increased IL‐2, IP‐10, IFN‐α, MIP‐1, MIP‐2, IL‐1α, IL‐1β, and RANTES expression (Figure S3c–k). While several inflammatory mediators had normalized by 4 months, others remained elevated or emerged later, including IP‐10 and RANTES expression and delayed increases in M‐CSF and IL‐13 (Figure S3d,e,k,l). Together, these data indicate a dynamic inflammatory response with both early and persistent components.

### 2.4 Irradiation Induces Hepatic Metabolic Dysfunction Together With Tissue‐Specific Senescence Responses in Liver and Skeletal Muscle

**FIGURE 4:**
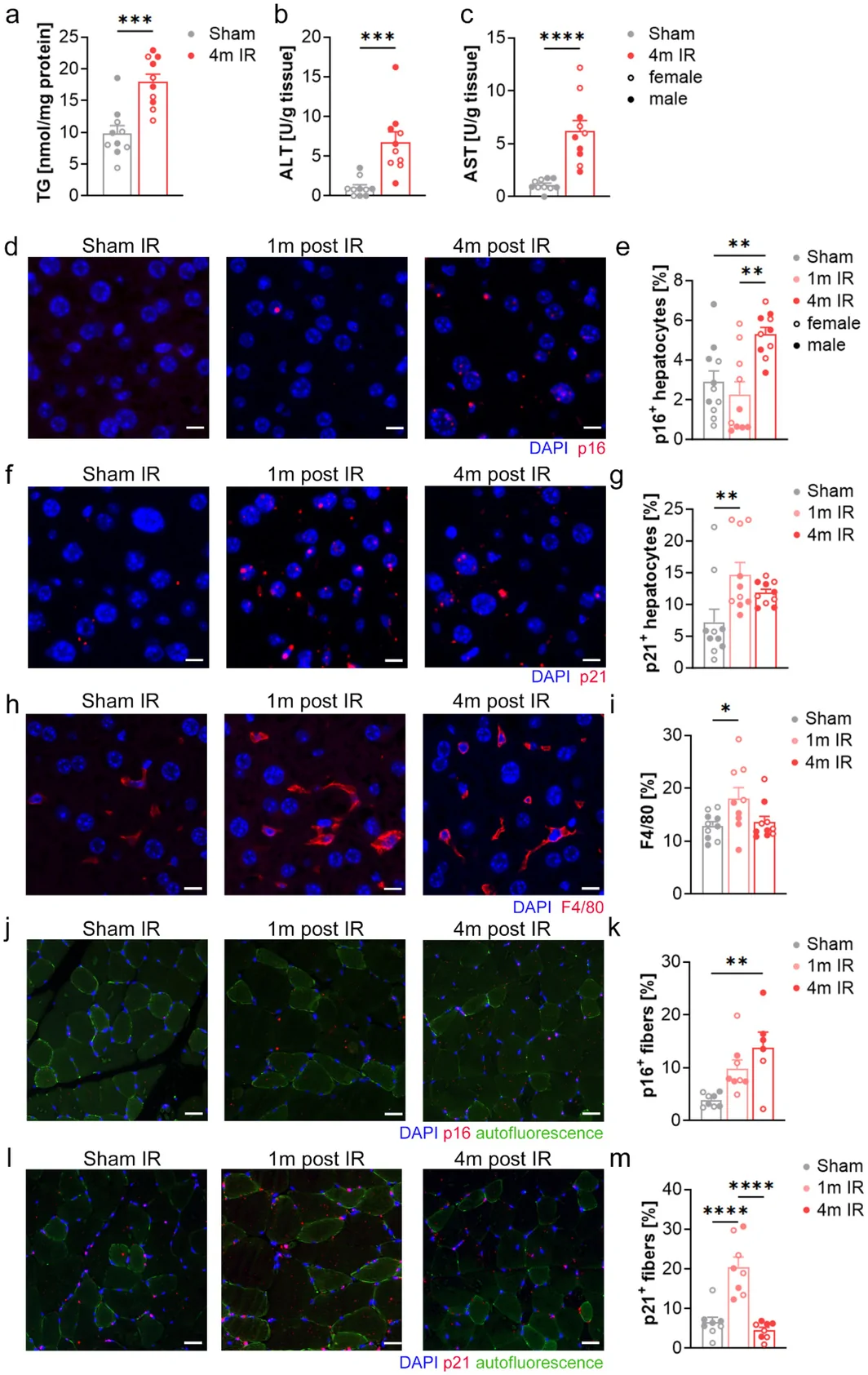
Irradiation induces hepatic metabolic alterations and tissue‐specific senescence responses in liver and skeletal muscle. (a) Hepatic triglyceride (TG) content in Sham and 4 months after irradiation (IR) mice (*p* = 0.0002). (b) Serum alanine aminotransferase (ALT) in Sham and 4 months after irradiation (IR) mice (*p* = 0.0004). (c) Aspartate aminotransferase (AST) in Sham and 4 months after irradiation (IR) mice (*p* < 0.0001). (d) Representative images of RNA‐ISH in hepatocytes stained for p16 (red) and DAPI (blue) in Sham, 1 month, and 4 months after irradiation. (e) Quantification of p16^Ink4a^‐positive hepatocytes. p16^Ink4a^ positivity was significantly increased at 4 months compared to Sham (*p* = 0.0092) and 1 month post IR (*p* = 0.0013). (f) Representative images of RNA‐ISH in hepatocytes stained for p21^Cip1^ (red) and DAPI (blue) in Sham, 1 month, and 4 months after irradiation. (g) Quantification of p21^Cip1^‐positive hepatocytes. p21^Cip1^ positivity was significantly increased at 1 month compared to Sham (*p* = 0.0092). (h) Representative images of hepatic macrophages stained for F4/80 (red) and DAPI (blue) in Sham, 1 month, and 4 months after irradiation. (i) Quantification of F4/80^+^ cells. F4/80‐positive cells were significantly increased at 1 month post IR compared to Sham (*p* = 0.0407). (j) Representative images of p16^Ink4a^‐positive (red) muscle fibers and DAPI (blue) in Sham, 1 month, and 4 months after irradiation. (k) Quantification of p16^Ink4a^‐positive fibers. p16‐positive cells were significantly increased at 4 months post IR compared to Sham (*p* = 0.0026). (l) Representative images of p21^Cip1^‐positive (red) muscle fibers and DAPI (blue) in Sham, 1 month, and 4 months after irradiation. (m) Quantification of p21^Cip1^‐positive fibers. p21‐positive cells were significantly increased at 1 month post IR compared to Sham (*p* < 0.0001) and decreased at 4 months (*p* < 0.0001). Scale bars: 10 μm in d, f, and h; 30 μm in j and l. Open circles represent females and closed circles represent males. Statistical analyses: ordinary one‐way ANOVA for all panels. Sample sizes: Sham *n* = 11; 1 month IR *n* = 10; 4 months IR *n* = 10 animals per group. Data are presented as mean ± SEM. *p < 0.05, **p < 0.01, ***p < 0.001, ****p < 0.0001.

To determine whether irradiation‐induced senescence extends beyond the brain, we next examined metabolic and senescence‐associated changes in the liver as well as senescence markers in skeletal muscle (Figure 4). Irradiated mice exhibited significant hepatic triglyceride accumulation together with increased serum alanine aminotransferase (ALT) and aspartate aminotransferase (AST) at 4 months post‐irradiation (Figure 4a–c), consistent with persistent metabolic dysfunction and liver injury.

Within the liver, p16^Ink4a^‐positive hepatocytes were significantly increased at 4 months but not at 1 month following irradiation (Figure 4d,e). In contrast, p21^Cip1^‐positive hepatocytes were already significantly increased at 1 month (Figure 4f,g), preceding the delayed accumulation of p16^Ink4a^. Irradiation also induced an early inflammatory response in the liver, with increased numbers of F4/80‐positive macrophages detected at 1 month (Figure 4h,i). Low‐magnification images confirmed the widespread distribution of senescence and inflammatory markers throughout the liver tissue (Figure S4a–c). Given recent reports describing p21‐expressing macrophages in the aged liver (Salladay‐Perez et al. 2026), we next examined p21 expression in macrophages following irradiation. p21/F4/80 double‐positive cells were increased at 1 month after irradiation (Figure S4h).

To further characterize the hepatic response, we evaluated γH2A.X foci and TAF. TAF‐positive hepatocytes were significantly increased at both 1 and 4 months after irradiation, with the highest levels observed at 1 month (Figure S4d,e). Likewise, both the average number of γH2A.X foci and the proportion of γH2A.X‐positive hepatocytes were elevated at 1 month before declining at 4 months, although TAF remained increased (Figure S4f,g). This is consistent with our previous findings that γH2A.X‐associated DNA damage can be repaired over time after genotoxic stress (Hewitt et al. 2012), whereas telomere‐associated DNA damage is largely irreparable and therefore persists (Fumagalli et al. 2012). Consistent with this early inflammatory response, p21/F4/80 double‐positive cells were also increased at 1 month following irradiation (Figure S4h).

Given the changes in frailty observed following irradiation, we next examined skeletal muscle as a tissue likely contributing to these functional outcomes. Skeletal muscle exhibited a temporal senescence response similar to that observed in the liver. p16^Ink4a^‐positive muscle fibers were significantly increased at 4 months after irradiation, whereas p21^Cip1^‐positive fibers increased at 1 month before declining at 4 months (Figure 4j–m). To determine whether senescence marker induction was accompanied by structural and metabolic changes, we quantified intramuscular triglyceride content and centrally nucleated fibers. We found that muscle triglyceride content was increased at 4 months after irradiation (Figure S4i), whereas a modest increase in centrally nucleated muscle fibers was observed at 4 months following irradiation, although this effect showed considerable variability across male animals (Figure S4j).

Collectively, these findings demonstrate that whole‐body irradiation induces tissue‐specific metabolic dysfunction together with a temporally structured senescence program characterized by early p21^Cip1^ induction, persistent DNA damage signaling and inflammation, followed by delayed accumulation of p16^Ink4a^‐positive cells across multiple tissues. These temporal differences may explain why senescent cell clearance initiated 1 month after irradiation was ineffective.

### 2.5 Delayed Senescent Cell Clearance Attenuates Irradiation‐Induced Functional Decline

**FIGURE 5:**
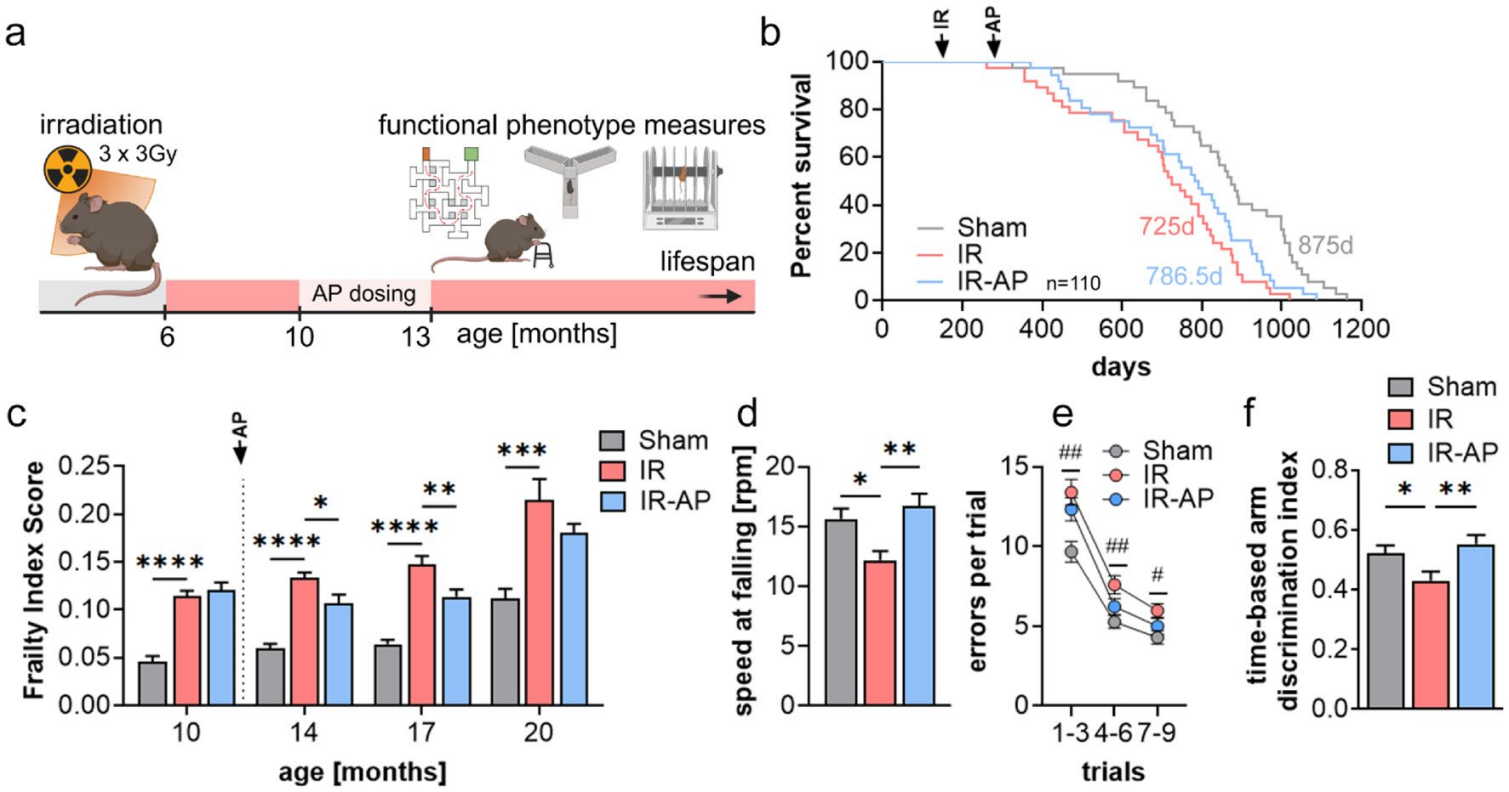
Late senolytic intervention attenuates irradiation‐induced functional decline. (a) Experimental design illustrating delayed AP treatment initiated 4 months (4 m) after irradiation with subsequent longitudinal assessment. (b) Kaplan–Meier survival curves of sham irradiated (Sham), irradiated (IR), and irradiated mice treated with AP (IR‐AP) following delayed intervention (combined sexes). IR vs. IR‐AP, *p* = 0.1668. Median survival: Sham 875 days, IR 725 days and IR‐AP 786.5 days. (c) Frailty index scores over time following delayed AP treatment. IR mice displayed increased frailty compared to Sham at all timepoints (*p* < 0.001). IR‐AP showed partial improvement compared to IR at 14 months (*p* = 0.0301) and 17 months (*p* = 0.0083), but not significantly at 10 or 20 months. (d) RotaRod performance expressed as speed at fall (rpm) following delayed treatment. IR vs. Sham, *p* = 0.0118; IR vs. IR‐AP, *p* = 0.0013. (e) Stone T‐maze performance shown as number of errors across trial blocks. IR mice performed worse than Sham (*p* = 0.0013–0.0172), whereas IR‐AP did not significantly differ from IR. (f) Y‐maze time‐based arm discrimination index following delayed treatment. IR vs. Sham, *p* = 0.0439; IR vs. IR‐AP, *p* = 0.0067. Statistical analyses: Survival curves were analyzed by log‐rank (Mantel‐Cox) test. Frailty and behavioral outcomes were analyzed by ordinary or two‐way ANOVA as indicated, followed by Tukey's or Dunnett's multiple comparisons tests. Sample sizes: (b) Sham *n* = 37 (16M/21F), IR *n* = 37 (17M/20F), IR‐AP *n* = 36 (17M/19F). (c) Sham *n* = 33–25, IR *n* = 33–19, IR‐AP *n* = 33–19 across timepoints. (d) Sham *n* = 32, IR *n* = 27, IR‐AP *n* = 26. (e) Sham *n* = 48, IR *n* = 48, IR‐AP *n* = 43. (f) Sham *n* = 40, IR *n* = 40, IR‐AP *n* = 38. Data are presented as mean ± SEM.*p < 0.05, **p < 0.01, ***p < 0.001, ****p < 0.0001.

The delayed accumulation of p16^Ink4a^‐positive cells in brain and liver prompted us to test whether senescent cell clearance initiated at a later timepoint would improve functional outcomes. We administered AP beginning 4 months after irradiation, coinciding with the time of robust p16^Ink4a^ induction, and then longitudinally monitored survival and functional parameters (Figure 5a).

In contrast to clearance initiated at 1 month, delayed AP treatment was associated with improved outcomes. Although overall survival in the combined cohort did not reach statistical significance compared with untreated irradiated mice, median survival increased from 725 days in irradiated mice to 786.5 days following delayed AP treatment (Figure 5b). Sex‐stratified analyses revealed a significant survival benefit in females, with median survival increasing from 696.5 to 743 days, whereas no detectable benefit was observed in males (Figure S5a,b). Notably, females also exhibited the greatest irradiation‐induced reduction in survival in Figure 2, consistent with delayed senescent cell clearance preferentially benefiting the sex most susceptible to irradiation‐induced mortality.

To determine whether irradiation or delayed senescent cell clearance affected gross physiological parameters at these time points, we assessed body and organ weights. Neither body weight nor liver and brain weights differed significantly between sham, irradiated, and AP‐treated irradiated mice at either 1 or 4 months post‐irradiation (Figure S5c–f).

Frailty progression was partially attenuated by delayed senescent cell clearance. Irradiated mice displayed elevated frailty scores compared with sham controls across time points, whereas AP‐treated irradiated mice exhibited reduced frailty at intermediate ages (Figure 5c). Sex‐stratified analyses indicated that this effect was primarily driven by females, with males showing no significant improvement (Figure S5g–i).

Motor performance was also improved following delayed intervention. RotaRod testing demonstrated reduced performance in irradiated mice compared with sham controls, whereas AP‐treated irradiated mice showed a significant improvement relative to untreated irradiated mice (Figure 5d). Similar benefits were observed in both sexes, although the pattern of impairment differed between males and females (Figure S5e,f).

Cognitive outcomes exhibited domain‐specific effects. In the Stone T‐maze, irradiated mice made more errors than sham controls and delayed AP treatment did not reduce error rates (Figure 5e). In contrast, delayed senescent cell clearance improved Y‐maze performance. Irradiated mice showed impaired time‐based arm discrimination compared with sham animals, and this deficit was significantly attenuated in AP‐treated mice (Figure 5f). This improvement was most pronounced in females (Figure S5g,h).

Collectively, these findings demonstrate that senescent cell clearance initiated after the establishment of robust p16^Ink4a^‐positive cell accumulation confers functional benefit, improving frailty progression, motor performance, and aspects of cognitive function. These results indicate that the therapeutic efficacy of p16^Ink4a^‐targeted senescent cell clearance depends on the timing of intervention following genotoxic stress.

### 2.6 Delayed p16^Ink4a^ ‐Positive Cell Clearance Attenuates Tissue Pathology Following Irradiation

Having established that delayed senescent cell clearance improves functional outcomes, we next investigated the tissue changes associated with these benefits, focusing initially on the brain.

**FIGURE 6:**
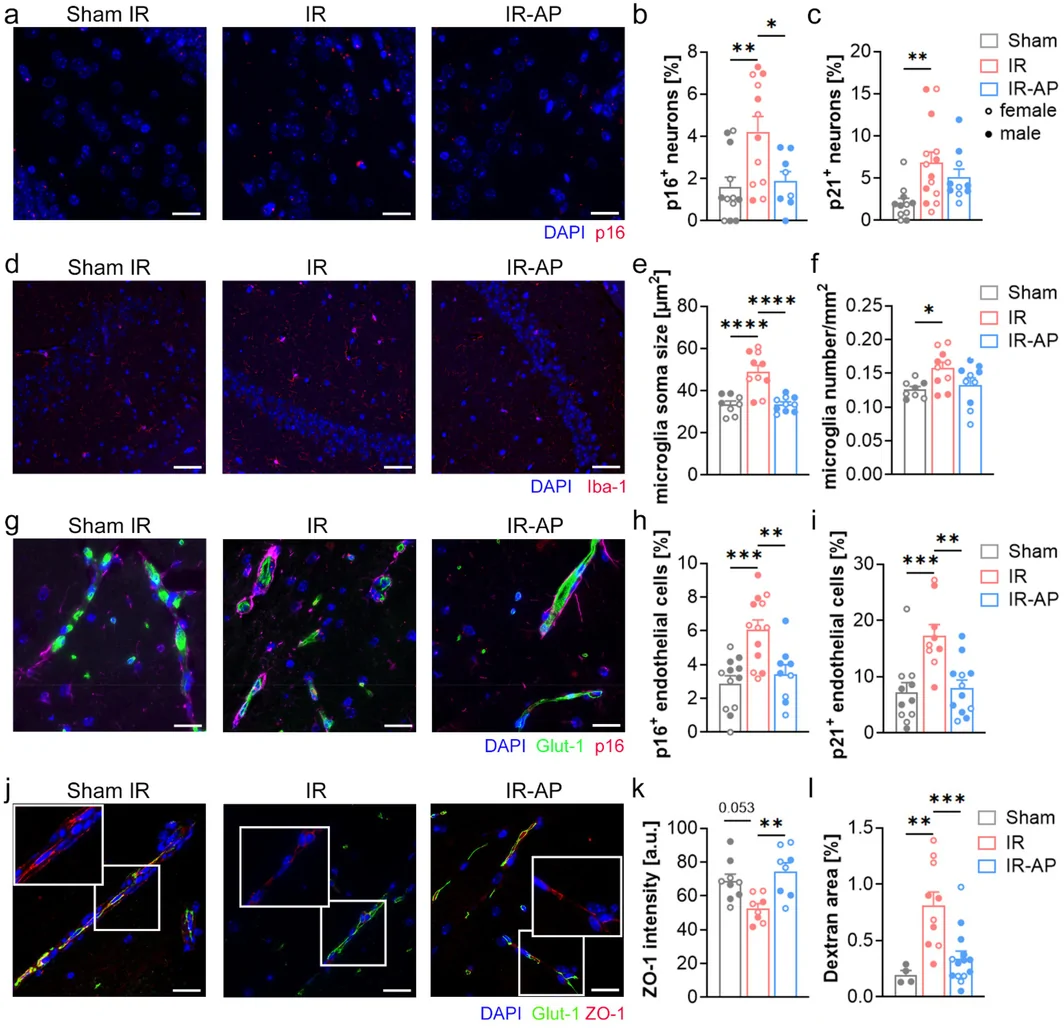
Late senolytic treatment modulates senescence, neuroinflammation, and blood–brain barrier integrity after irradiation. (a) Representative images of RNA‐ISH in hippocampal neurons stained for p16 (red) and DAPI (blue) in sham IR (Sham), irradiated with vehicle treatment (IR), and irradiated with AP treatment (IR‐AP). (b) Quantification of p16^Ink4a^ neurons in the hippocampus. Irradiation increased p16^Ink4a^ neurons compared to Sham (*p* = 0.0043), and AP treatment reduced p16^Ink4a^ neurons compared to IR (*p* = 0.0228). (c) Quantification of p21^Cip1^ neurons in the hippocampus. Irradiation increased p21^Cip1^ neurons compared to Sham (*p* = 0.0050) but no significant changes were observed with AP (IR vs. IR‐AP, *p* = 0.4506). (d) Representative images of hippocampal microglia stained for Iba‐1 (red) and DAPI (blue). (e) Quantification of microglial soma size. Irradiation increased microglial soma size compared to Sham (*p* < 0.0001), which was significantly reduced by AP treatment (*p* < 0.0001 vs. IR). (f) Microglial density (number/mm^2^). IR mice exhibited increased microglial numbers compared to Sham (*p* = 0.0300), while IR‐AP did not significantly differ from IR (*p* = 0.0822). (g) Representative images of RNA‐ISH in cortical endothelial cells stained for p16 (red), Glut‐1 (green), and DAPI (blue). (h) Quantification of p16^+^ endothelial cells. Irradiation increased p16^Ink4a^ endothelial cells compared to Sham (*p* = 0.0002), and AP treatment significantly reduced this increase (*p* = 0.0030 vs. IR). (i) Quantification of p21^Cip1^ endothelial cells. Irradiation increased p21^Cip1^ endothelial cells compared to Sham (*p* = 0.0007), and AP treatment reduced p21^Cip1^ endothelial cells (*p* = 0.0015 vs. IR). (j) Representative cortical vessel images stained for ZO‐1 (red), Glut‐1 (green), and DAPI (blue). The larger inset shows a 150% magnification of the boxed region; Glut‐1 was omitted in the magnification to better visualize ZO‐1. (k) Quantification of ZO‐1 intensity. Irradiation reduced ZO‐1 intensity compared to Sham (*p* = 0.0207), and AP treatment restored ZO‐1 levels (*p* = 0.0334 vs. IR). (l) Quantification of dextran extravasation as a measure of blood–brain barrier permeability. Irradiation increased dextran leakage compared to Sham (*p* = 0.0023), which was reduced by AP treatment (*p* = 0.0009 vs. IR). Open circles represent females and closed circles represent males. Scale bars: 20 μm. Statistical analyses were performed using one‐way ANOVA followed by Dunnett's multiple comparisons test. Sample sizes: *n* = 8–13 animals per group. Data are presented as mean ± SEM. *p < 0.05, **p < 0.01, ***p < 0.001, ****p < 0.0001.

In the hippocampus, irradiation increased the number of both p16^Ink4a^ ‐and p21^Cip1^‐positive neurons. Delayed AP treatment significantly reduced p16^Ink4a^ positivity but had no effect on neuronal p21^Cip1^ expression (Figure 6a–c, Figure S6a). Delayed senescent cell clearance was also associated with reduced neuroinflammation. Irradiation increased both microglial density and soma size, indicative of microgliosis and activation, whereas AP treatment significantly reduced microglial hypertrophy without affecting microglial density (Figure 6d–f). AP treatment also reduced the number of IL‐6‐positive neurons but did not alter IL‐1α expression (Figure S6b,c). Finally, hippocampal neuronal TAF were increased following irradiation and significantly reduced after AP treatment (Figure S6d).

A similar pattern was observed in the cortex. Irradiation increased the number of p16^Ink4a^‐positive GLUT1+ endothelial cells, whereas delayed AP treatment significantly reduced their abundance (Figure 6g,h). p21^Cip1^ positivity in GLUT1+ endothelial cells was likewise increased following irradiation and reduced by AP treatment (Figure 6i, Figure S6e).

To determine whether reduced endothelial senescence was accompanied by improved blood–brain barrier (BBB) integrity, we next examined endothelial tight junctions and vascular permeability. Irradiation reduced cortical ZO‐1 intensity, consistent with disruption of endothelial tight junctions, whereas AP treatment restored ZO‐1 levels (Figure 6j,k). Functional assessment using dextran extravasation demonstrated increased BBB permeability following irradiation, which was significantly reduced by delayed senescent cell clearance (Figure 6l).

Additional analyses of the cerebral cortex showed that irradiation increased both p16^Ink4a^‐ and p21^Cip1^‐positive GFAP+ astrocytes, whereas AP treatment selectively reduced astrocytic p16^Ink4a^ positivity (Figure S6f,g).

We next investigated the effects of delayed senescent cell clearance in the liver and skeletal muscle.

In the liver, AP treatment reduced hepatic triglyceride accumulation and decreased serum ALT and AST levels, consistent with improved liver health following irradiation (Figure S6h–j). These changes were accompanied by fewer p16^Ink4a^‐positive hepatocytes and a lower proportion of γH2A.X‐ and TAF‐positive hepatocytes, whereas hepatic p21^Cip1^ expression remained unchanged (Figure S6k–n). In skeletal muscle, AP treatment did not affect triglyceride content or the number of p16^Ink4a^‐positive fibers, despite a significant reduction in p21^Cip1^‐positive fibers (Figure S6o–q).

Together, these findings demonstrate that delayed senescent cell clearance attenuates irradiation‐induced tissue pathology in the brain and liver, consistent with the improved physiological function observed after delayed intervention.

## 3 Discussion

Radiation therapy remains a cornerstone of cancer treatment and is essential for cure in most malignancies. Individuals who survive cancer long‐term develop levels of frailty comparable to those seen in the general population several decades older, effectively advancing frailty onset by approximately 30 years (Ness et al. 2013). Accelerated frailty, increased disease burden, and higher mortality observed in cancer survivors are consistent with a phenotype of treatment‐associated premature aging (Demaria et al. 2017; Ness et al. 2018). At present, there are no effective interventions to prevent or reverse this long‐term consequence of irradiation.

Accumulation of senescent cells following cancer therapies has been proposed as a mechanistic driver of this accelerated aging‐like phenotype. Multiple studies have now shown that targeting senescent cells can mitigate specific consequences of cancer therapy (Prasanna et al. 2021). Senolytic interventions administered after sublethal irradiation, or following treatment with doxorubicin, have been reported to partially restore immune function, reduce bone loss, improve cardiac performance and physical activity, and limit liver damage (Demaria et al. 2017; Baar et al. 2017; Chandra et al. 2022; Palacio et al. 2019; Chang et al. 2016). More recently, Fielder et al. demonstrated that male mice irradiated and treated transiently with senolytic drugs, navitoclax or D + Q, exhibited reduced frailty progression and sustained improvements in short‐term memory (Fielder et al. 2022). While these studies support a role for senescent cells in therapy‐induced acceleration of aging processes, critical questions remain unresolved. In particular, the long‐term impact of senolytic interventions on survival trajectories after irradiation has not, to our knowledge, been systematically examined, and the optimal timing of intervention relative to the emergence of senescent cell burden remains unclear. Moreover, most pharmacologic senolytics lack strict cellular and senotype specificity, making it difficult to define which senescent cell populations are being targeted and which are responsible for the observed benefits.

Our study addresses these gaps directly. By evaluating both healthspan and survival, we determine whether p16^Ink4a^‐positive senescent cell clearance after irradiation influences not only functional decline but also long‐term survival. Importantly, we explicitly compare the timing of intervention. We show that clearance of p16^Ink4a^‐positive cells initiated 1 month after irradiation fails to improve survival or functional outcomes. This lack of detectable effect coincides with the absence of a substantial expansion of p16^Ink4a^‐positive cells at this early timepoint, despite robust induction of p21^Cip1^ and persistent DNA damage. In contrast, by 4 months after irradiation, p16^Ink4a^‐positive cells accumulate across multiple tissues, including the brain, liver, and skeletal muscle. Delayed clearance initiated at this stage improves frailty progression, neuromuscular performance, and cognitive function, indicating that the therapeutic efficacy of p16‐directed senescent cell clearance depends on the temporal establishment of a p16^Ink4a^‐positive senescent cell burden.

Delayed removal of p16‐positive cells also improved survival in female mice, increasing median survival by approximately 7% relative to irradiated controls. Although the combined‐sex analysis did not reach statistical significance, the rightward shift of the survival curve in females suggests a survival benefit that persists long after treatment cessation. Notably, irradiation produced a substantially greater reduction in survival in females than in males, indicating that the magnitude of senolytic benefit may scale with the severity of irradiation‐induced injury. In this context, the observed delay in mortality represents a meaningful extension of post‐irradiation survival.

Interestingly, the beneficial effects of delayed p16‐directed senescent cell clearance on survival were most apparent in female mice, despite males exhibiting greater impairment in several functional assays after irradiation. The mechanisms underlying these sex‐specific responses remain unclear but are consistent with increasing evidence that cellular senescence and tissue responses to genotoxic stress are influenced by biological sex. As the present study was not designed or powered to define sex‐specific mechanisms, future studies will be required to determine whether differences in senescent cell accumulation, tissue vulnerability, or immune responses underlie these observations.

Mechanistically, we observed accumulation of p16^Ink4a^‐positive cells in hippocampal neurons, cortical endothelial cells, astrocytes, and hepatocytes, accompanied by persistent neuroinflammatory changes, blood–brain barrier disruption, and hepatic metabolic dysfunction. Clearance of these cells reduced neuroinflammatory markers, decreased telomere‐associated DNA damage foci, improved endothelial tight junction integrity, restored blood–brain barrier function, and ameliorated hepatic triglyceride accumulation and liver injury. These findings align with prior studies showing that removal of senescent cells improves blood–brain barrier function and cognitive outcomes during aging (Novo et al. 2024; Costa et al. 2026), and suggest that persistent senescence‐associated inflammation is a key driver of post‐irradiation tissue dysfunction. Notably, the benefits of delayed clearance persisted long after cessation of AP treatment. This sustained improvement suggests that senescent cells act as initiating drivers of downstream tissue dysfunction rather than merely reflecting ongoing damage, and that their removal can durably alter the trajectory of post‐irradiation aging processes.

Importantly, p16‐positive cells represent only one senescent subtype. Emerging evidence indicates that p16‐ and p21‐expressing cells constitute distinct senescent populations with divergent biological roles (Saul et al. 2025; Sturmlechner et al. 2021). For example, radiation‐induced bone loss has been shown to be driven predominantly by p21‐positive cells (Chandra et al. 2022), underscoring tissue‐specific contributions of different senescence programs. Our findings demonstrate that selective removal of p16‐positive cells is sufficient to improve frailty, cognition, and survival after irradiation, but they do not exclude contributions from additional senescent subtypes. Consistent with this concept, delayed p16‐directed senescent cell clearance selectively reduced p16‐associated pathology while leaving several p21‐associated responses unchanged, further supporting the existence of temporally and biologically distinct senescent populations after irradiation. Together, these data support a model in which distinct senotypes emerge with different temporal dynamics and contribute to radiation‐induced dysfunction in a tissue‐ and context‐dependent manner. Our findings further suggest that these temporally distinct senescence programs have different therapeutic windows, with early p21‐associated responses reflecting an acute stress response, whereas later p16^Ink4a^‐positive cells represent the pathogenic population that becomes amenable to selective senolytic intervention.

From a translational perspective, these findings suggest that temporally targeted senolytic interventions may represent a viable strategy to mitigate long‐term functional decline in cancer survivors. Rather than immediate post‐therapy administration, our data support the concept that senolytic treatment may need to be aligned with the biological emergence of senescent cells to achieve maximal benefit.

Our results differ from a previous study on when senolytic treatment should be given. We found that removing p16‐positive cells 1 month after irradiation did not improve function, whereas that study reported benefits when treatment was given early using drugs such as Navitoclax or Dasatinib plus Quercetin (Fielder et al. 2022). This difference likely reflects the types of cells targeted. Pharmacological senolytics remove a broad range of senescent cells, while the *INK‐ATTAC* model removes only p16‐expressing cells. These findings highlight that both the type of senescent cells and the timing of intervention influence therapeutic outcomes.

Several limitations should be considered. Although the *INK‐ATTAC* model enables precise and controlled elimination of p16‐expressing cells, it targets only a subset of senescent cells and therefore may not capture the full diversity of senescence states present after irradiation. Distinct senotypes with different molecular programs and tissue distributions may contribute to pathology and may respond differently to interventions. In addition, translation to cancer survivors will require careful evaluation of tumor control, immune consequences, and optimal treatment timing in clinically relevant settings.

Together, our findings establish that irradiation‐induced senescence is a temporally dynamic process in which distinct senescence programs emerge sequentially across multiple tissues. Therapeutic efficacy depends critically on aligning senolytic intervention with the establishment of a pathogenic p16Ink4a‐positive senescent cell burden, providing a conceptual framework for temporally precise senolytic strategies in therapy‐associated premature aging.

## 4 Methods

### 4.1 Experimental Model

Experimental procedures were approved by the Institutional Animal Care and Use Committee (IACUC) at Mayo Clinic (protocol A00003365, A00008060). Two independent mouse cohorts were used in this study. Wild‐type C57BL/6J mice were used to characterize the temporal progression of irradiation‐induced senescence following whole‐body irradiation (Figures 1, 2, 3, 4). INK‐ATTAC mice were used to investigate the effects of genetic clearance of p16^Ink4a^‐positive senescent cells following early or delayed AP20187 treatment (Figures 5 and 6). INK‐ATTAC mice (both sexes) were bred at Mayo Clinic, aged to 5–8 months of age, and housed in a pathogen‐free facility under controlled environmental conditions, including a temperature range of 23°C–24°C and a 12‐h light/dark cycle. Mice were maintained in static, autoclaved HEPA‐ventilated microisolator cages (27 × 6.5 × 15.5 cm) containing autoclaved Enrich‐o'Cobs bedding. Bedding and cages were changed biweekly in Class II biosafety cabinets. Routine quarterly pathogen testing consistently yielded negative results.

For the longitudinal phenotypic studies (survival, frailty, neuromuscular function, cognitive testing, and lifespan analyses; Figures 1, 2, and 5), mice were irradiated using a ^137Cs^γ‐irradiation source. As tissues from these cohorts were not available for subsequent molecular analyses, independent cohorts of irradiated C57BL/6J and INK‐ATTAC mice were established for tissue characterization (Figures 3, 4, and 6). These mechanistic studies were performed using X‐ray irradiation. Importantly, comparable induction of senescence markers and tissue pathology was observed following both irradiation modalities.

5–7‐month‐old male and female mice were subjected to fractionated total body irradiation (TBI). During exposure, mice were placed in a rotating plastic container to ensure uniform whole‐body radiation delivery. Animals received 3 × 3–3.2 Gy TBI administered every third day (Tuesday, Friday, and Monday). To reduce the risk of infection, mice received 1% Baytril (enrofloxacin) in drinking water, starting 2 days prior to the first irradiation and continuing until 14 days after the final irradiation dose. Antibiotic‐containing water bottles were protected from light and replaced every second day.

For senescent cell clearance, INK‐ATTAC mice were administered AP20187 (10 mg/kg body weight) by intraperitoneal injection. AP20187 was dissolved in a vehicle consisting of 4% ethanol, 10% PEG‐400, and 2% Tween‐20 in distilled water. Control animals received the corresponding vehicle solution by intraperitoneal injection. Treatment was administered in cycles consisting of three consecutive days of injections (Monday–Wednesday), followed by a recovery period without treatment for the remainder of the week and the subsequent week. A second treatment cycle was initiated in week 3. For early intervention, treatment was initiated 1 month after irradiation and consisted of one two‐cycle course as described above. For late intervention, treatment was initiated 4 months after irradiation. Mice received two treatment rounds: the first at 4 months post‐irradiation and a second identical round at 6 months post‐irradiation.

At the end of the treatment period, mice were euthanized, and organs were harvested for downstream analyses. Mouse tissues were either snap‐frozen in liquid nitrogen for biochemical analysis or fixed in 4% paraformaldehyde (PFA) for 24 h. Fixed samples were then processed, embedded in paraffin, and sectioned at 3 μm thickness for further analysis.

### 4.2 Y‐Maze

Spatial and working memory assessments in mice were conducted using a Y‐maze setup. The Y‐maze apparatus consists of three identical arms with elevated walls. Mice were habituated to the testing room for at least 1 h prior to the experiment. During the training phase, one arm (the novel arm) was blocked, and mice explored the other two arms for 10 min. Following a 60‐min rest period, the test phase was conducted, during which all three arms were accessible for 5 min. Behavior was recorded with a camera and analyzed manually. Cognitive performance was evaluated by measuring spontaneous alternation, specifically the number of entries into the novel arm and the total time spent in it.

### 4.3 Stone T‐Maze

Cognitive performance was assessed using a water‐motivated version of the Stone T‐maze, custom‐built by the Mayo Clinic workshop, which is sensitive to age‐related deficits in learning and memory. The apparatus consisted of an acrylic‐roofed maze positioned in a shallow steel pan filled with water, motivating mice to navigate toward a dry goal box while keeping their heads above water. On Day 1, mice underwent straight‐run training to acclimate to the escape paradigm. Each mouse was placed in the start box and allowed to traverse a straight maze to reach the goal box. Upon entry, mice were gently dried with a towel and placed in a heated holding cage. The testing room was maintained at a comfortable ambient temperature, and the holding cage was partially warmed with a heat lamp, allowing self‐regulated exposure to heat. Each mouse completed three straight‐run trials per session, with three sessions separated by 15‐min intervals, totaling nine training trials. Maze testing was conducted the following day. After 1 h of habituation in the testing room, each mouse was placed in the start box and allowed to explore the maze for up to 6 min. Latency to reach the goal box and the number of errors were defined as complete mouse head entries into incorrect arms. Inter‐trial intervals of 15 min were maintained. After each trial, mice were dried with a towel and returned to the heated holding cage. Trials in which a mouse failed to reach the goal box within 5 min were marked as unsuccessful. Mice exhibiting three consecutive unsuccessful trials were excluded from further testing. Each mouse completed a total of nine acquisition trials.

### 4.4 RotaRod

Motor coordination and maximal walking speed were assessed using an accelerating RotaRod Rotamex 5 (Columbus Instruments). Mice were trained for two consecutive days prior to testing on Day 3. Training sessions consisted of mice remaining on the rotating rod on an accelerated rotating rod for up to 300 s on Days 1 and 2, respectively. On the test day, the rod accelerated linearly from 4 to 40 rpm over 300 s. The latency to fall and the corresponding speed at the time of falling were recorded. Each mouse completed three test trials, and the average performance across trials was used for analysis.

### 4.5 Hanging (Wire) Test

Neuromuscular strength and endurance were assessed using the wire hanging test. Male and female mice were placed with their forelimbs at the center of a horizontally suspended metal wire (2 mm diameter, ~30 cm length). Once the mouse securely grasped the wire, it was gently released and allowed to hang freely. Each trial lasted a maximum of 60 s. Successful performance on the first trial was defined by one of the following criteria: (i) lifting all four limbs onto the wire, (ii) reaching either end of the wire (latency recorded), or (iii) maintaining grip for the full 60‐s duration. Each mouse underwent three trials within a 15‐min testing period. The wire was suspended 39 cm above the testing surface. Soft bedding was placed underneath to prevent injury in case of falling. This height was sufficient to discourage voluntary dropping while ensuring animal safety.

### 4.6 Forelimb Grip Strength Analysis

Forelimb grip strength was assessed using a grip strength meter equipped with a computer‐integrated force transducer. Mice were allowed to grasp a horizontal metal bar with their forelimbs and were gently pulled backward in a horizontal plane until they released the bar. The peak force generated during each trial was recorded. Each mouse performed three consecutive trials, and the mean peak force was used for statistical analysis.

### 4.7 Frailty Measurements

Frailty was evaluated using a 30‐parameter clinical frailty index, as previously described by Whitehead et al. (2014). This method was standardized and described in our paper (Fielder et al. 2022; Baar et al. 2017). Each parameter was scored on a three‐point scale: 0 for absent, 0.5 for mild, and 1 for severe phenotype. Assessed parameters included evaluations of the integument, musculoskeletal system, vestibulocochlear/auditory function, ocular and nasal systems, digestive system, urogenital system, respiratory system, signs of discomfort, as well as measurements of body weight (g) and body surface temperature (°C). This index provides a non‐invasive, quantitative measure of overall health status in aging mice. The frailty index was calculated as the average score across all parameters assessed.

### 4.8 Cytokine Array

Cytokine levels in hippocampal homogenates were quantified using a multiplex immunoassay platform. Tissue samples were homogenized in lysis buffer containing protease inhibitors and centrifuged to remove debris. Supernatants were collected, and total protein concentration was determined using the Bio‐Rad protein assay. Samples were then submitted to Eve Technologies Corporation (Calgary, Alberta, Canada) for analysis using the Mouse Cytokine/Chemokine 18‐Plex Discovery Assay (MDHSTC18) platform. The assay was performed according to the company's standard protocols. The assay measured a total of 18 cytokines/chemokines; however, only analytes that were reliably detected were included in the analyses and are reported in this manuscript. Cytokine concentrations were calculated from standard curves and are expressed in pg/mL. All values were normalized to total protein content and reported relative to control groups.

### 4.9 Tissue Section Immunostaining

Formalin‐fixed, paraffin‐embedded (FFPE) tissue sections (3 μm) were deparaffinized in HistoClear and rehydrated through a graded ethanol series: 100% ethanol, 90% ethanol, and 70% ethanol, followed by two washes in distilled water. Antigen retrieval was performed by boiling slides in citrate buffer (pH 6.0; Agilent‐Dako, S236984) for 10 min, followed by cooling at room temperature for 20 min and two PBS washes (5 min each). Sections were blocked in 1% bovine serum albumin (BSA), 1:60 normal goat serum or fetal bovine serum in PBS for 30 min at room temperature, then incubated overnight at 4°C with the following primary antibodies: rabbit anti‐Iba1, rabbit anti‐GLUT1, mouse anti‐ZO‐1 and guinea pig anti‐GFPA. After washing with PBS, sections were incubated with species‐specific secondary antibodies for 1 h at room temperature. Following three PBS washes, slides were mounted with ProLong Gold Antifade Mountant containing DAPI (Invitrogen) for nuclear counterstaining. For all immunohistochemical analyses including RNA‐ISH and Immuno‐FISH, quantification parameters depended on the specific marker assessed. For tissue‐based senescence markers (e.g., p16, p21, TAF), a minimum of 100 cells per animal were analyzed. The number of fields examined was kept consistent across animals and experimental groups for each staining. The total number of images acquired per animal depended on the magnification used during imaging (e.g., 20× or 40× for general tissue analysis and 63× for TAF quantification).

### 4.10 RNA‐ISH

RNA in situ hybridization was performed using the RNAscope 2.5 HD Reagent Kit‐RED (Advanced Cell Diagnostics, ACD) according to the manufacturer's protocol. Formalin‐fixed, paraffin‐embedded (FFPE) tissue sections were deparaffinized in HistoClear (2 × 5 min), rehydrated in 100% ethanol (2 × 1 min), and air‐dried. Sections were incubated with hydrogen peroxide (H_2_O_2_) for 10 min at room temperature, followed by two washes in distilled water. Antigen retrieval was performed by boiling slides in 1X RNAscope Target Retrieval solution for 15 min. After rinsing in distilled water and 100% ethanol, slides were air‐dried and incubated with Protease Plus reagent for 30 min at 40°C. Tissue sections were then hybridized with target‐specific probes against p21 (ACD #408551), p16 (ACD #411011), IL‐6 (ACD #315891), and IL‐1a (ACD #440391) for 2 h at 40°C. Signal amplification was carried out sequentially using AMP1 (30 min at 40°C), AMP2 (15 min at 40°C), AMP3 (30 min at 40°C), AMP4 (15 min at 40°C), AMP5 (30 min at room temperature), and AMP6 (15 min at room temperature), with ACD wash buffer (WB) used for intermediate washes between steps. Chromogenic detection was performed using the RNAscope 2.5 HD Reagent Kit‐RED. Following five final washes in distilled water, slides were counterstained and mounted using ProLong Gold Antifade Mountant with DAPI (Invitrogen). RNA‐ISH quantification was performed by manually scoring positive cells relative to the total number of cells in each field. Nuclei were first identified and counted based on DAPI staining to determine the total cell number. Cells were classified as RNA‐ISH–positive if they exhibited one or more foci corresponding to p16, p21, IL‐6, and IL‐1a transcripts. The percentage of RNA‐ISH‐positive cells was calculated as the ratio of positive cells to total DAPI‐positive cells.

### 4.11 Immuno‐FISH

Formalin‐fixed, paraffin‐embedded (FFPE) tissue sections were deparaffinized in 100% HistoClear and rehydrated through a graded ethanol series (100%, 90%, and 70%; 2 × 5 min each), followed by two 5‐min rinses in distilled water. Antigen retrieval was performed by immersing slides in 0.01 M citrate buffer (pH 6.0) and heating to the boiling point for 10 min. After cooling to room temperature, sections were rinsed in distilled water for 5 min. Tissue sections were blocked using normal goat serum (1:60 in PBS with 1% BSA) for 30 min at room temperature, followed by additional blocking with an Avidin/Biotin Blocking Kit (Vector Laboratories, Burlingame, CA) for 15 min per step. Sections were then incubated overnight at 4°C with a rabbit monoclonal anti‐γH2AX antibody (1:200; Cell Signaling Technology, #9718). Following three PBS washes, sections were incubated with a biotinylated goat anti‐rabbit secondary antibody (1:200; Vector Laboratories, PK‐6101) for 30 min at room temperature. After three more PBS washes, fluorescein‐conjugated Streptavidin‐Cy5 (1:500; Vector Laboratories, A‐2011) was applied for 30 min at room temperature. Slides were washed again in PBS (3 × 5 min), then post‐fixed with 4% paraformaldehyde in PBS for 20 min to stabilize antibody complexes. Sections were subsequently dehydrated in a cold ethanol gradient (70%, 90%, and 100%; 3 min each) and air‐dried. For telomere‐specific peptide nucleic acid fluorescence in situ hybridization (PNA‐FISH), 10 μL of hybridization solution, containing 70% deionized formamide, 20 mM MgCl_2_, 1 M Tris (pH 7.2), 5% blocking reagent, and 2.5 μg/mL Cy3‐labeled (CCCTAA) telomere‐specific PNA probe (PANAGENE), were applied to each section. Slides were denatured at 80°C for 10 min and hybridized for 2 h at room temperature in the dark. Post‐hybridization washes included 70% formamide in 2× SSC (10 min), followed by 2× SSC (10 min) and PBS (10 min). Slides were mounted with ProLong Gold Antifade Mountant with DAPI (Invitrogen) and imaged using high‐resolution z‐stacked fluorescence microscopy with a 63× objective lens. For telomere‐associated foci (TAF) quantification, z‐stack images were acquired to visualize DNA damage foci in three dimensions (as we have done before (Hewitt et al. 2012)). TAF were manually quantified by first identifying γ‐H2AX foci within each nucleus, followed by detection of telomeric signals using a telomere‐specific probe. A TAF was identified when a γ‐H2AX foci colocalized with a telomeric signal throughout the z‐stack. The total number of TAFs per nucleus was subsequently recorded.

### 4.12 Microscopic Imaging

Microscopic imaging was performed using a Leica DFC7000GT inverted microscope.

### 4.13 Quantification and Statistical Analysis

All data are presented as mean ± standard error of the mean (SEM), unless otherwise indicated. Sample sizes were determined based on prior experience with similar experimental paradigms and published studies; no formal power calculations were performed. Animals were randomly assigned to experimental groups where applicable. Investigators were blinded to group allocation during data collection and analysis whenever feasible. Inclusion and exclusion criteria were defined prior to experimentation, and no data were excluded except in cases of predefined technical failure (e.g., assay failure or sample loss).

Statistical analyses were performed using GraphPad Prism version 10 (GraphPad Software, San Diego, CA). Data distribution was assessed prior to selection of parametric or non‐parametric tests. Survival curves were analyzed using the log‐rank (Mantel‐Cox) test, with Gehan‐Breslow‐Wilcoxon test applied where indicated. Frailty progression over time was assessed by linear regression with comparison of slopes. Behavioral and molecular endpoints were analyzed using unpaired two‐tailed Student's *t*‐tests (with Welch's correction where appropriate), one‐way or two‐way analysis of variance (ANOVA), or mixed‐effects models for repeated measures, followed by Tukey's, Dunnett's, or Šídák's multiple comparisons tests as specified in the respective figure legends. Categorical data were analyzed using Fisher's exact test.

Exact sample sizes (*n*), statistical tests, and corresponding *p*‐values are reported in the figure legends. A *p*‐value < 0.05 was considered statistically significant. Significance thresholds are denoted as follows: *p < 0.05; **p < 0.01; ***p < 0.001; ****p < 0.0001.

## Author Contributions

Diana Jurk and James L. Kirkland conceived and supervised the study. Diana Jurk interpreted the data and wrote the manuscript. Karla Valdivieso, Melanie Weigand, Daniela G. Costa, Nick Pirius, Helene Martini, Shivangi Oberai, and Christina Inman performed experiments and contributed to data acquisition and/or analysis. Tamara Tchkonia contributed to data interpretation. Thomas von Zglinicki, Yi Zhu, Sundeep Khosla, NKL, João F. Passos, and James L. Kirkland provided critical revisions and important intellectual input to the manuscript.

## Funding

This work was supported by National Institute on Aging (P01AG062413) (Diana Jurk, Sundeep Khosla, James L. Kirkland, Nathan LeBrasseur, João F. Passos), R01AG068182 (Diana Jurk), R01AG086085 (Sundeep Khosla), Hevolution HR‐GRO‐23‐1199144‐8 (Sundeep Khosla), Hevolution/AFAR (Diana Jurk), UG3/UH3CA268103 (João F. Passos); R01AG068048 (João F. Passos); R01AG82708 (João F. Passos); the Glenn Foundation for Medical Research (João F. Passos and NKL); HF‐GRO‐23‐1199262‐27 (Yi Zhu); R01AG087387 (Yi Zhu); R37AG13925 (James L. Kirkland and Tamara Tchkonia); R33AG061456 (James L. Kirkland and Tamara Tchkonia); the Connor Fund (James L. Kirkland and Tamara Tchkonia); Robert J. and Theresa W. Ryan (James L. Kirkland and Tamara Tchkonia); and the Noaber Foundation (James L. Kirkland and Tamara Tchkonia). Helene Martini and Gung Lee were supported by the Robert and Arlene Kogod Center on Aging Career Development Award.

## Disclosure

Permission Statement: No previously published material has been used in this manuscript. Figures were created by the authors (some using BioRender.com). Graphics: Schematic illustrations for experimental design in Figures 1a, 2a and 5a were created with BioRender.com.

## Conflicts of Interest

Patents about the *INK‐ATTAC* mouse model are held by James L. Kirkland, Tamara Tchkonia, Jan van Deursen, Darren Baker, and Mayo Clinic. This research was reviewed by the Mayo Clinic Conflicts of Interest Review Board and conducted in compliance with Mayo Clinic policy.

## Supporting information


**Figure S1:** Sex‐stratified data for whole‐body irradiation induces long‐term functional decline and cognitive impairment.
**Figure S2:** Early senolytic intervention does not significantly prevent functional decline following irradiation.
**Figure S3:** Time‐dependent neuroinflammatory changes in the hippocampus following whole‐body irradiation.
**Figure S4:** Additional characterization of irradiation‐induced hepatic damage, inflammation, lipid accumulation, and skeletal muscle pathology.
**Figure S5:** Sex‐stratified functional and cognitive outcomes following delayed (4 m) AP treatment after irradiation.
**Figure S6:** Delayed p16^Ink4a^ clearance attenuates irradiation‐induced tissue pathology.

## Data Availability

The data supporting the findings of this study are available from the corresponding author upon reasonable request. No large‐scale datasets were generated or analyzed in this study.
