# Supplementary material for: Timing‐Dependent Clearance of p16‐Positive Cells Mitigates Radiation‐Induced Accelerated Aging
Source: Aging Cell. 2026 Aug 26;25(9):e70693. doi: 10.1111/acel.70693 (PMC13519039; doi:10.1111/acel.70693)
Supplement: Supplementary file 1 [file ACEL-25-e70693-s001.docx]

**Figure S1. Sex-stratified data for whole-body irradiation induces long-term functional decline and cognitive impairment.**

**a)** Kaplan-Meier survival curves of sham-treated (Sham) and irradiated (IR) male mice. Irradiation significantly reduced survival in males (P = 0.0448; Gehan-Breslow-Wilcoxon P = 0.2022). Median survival: Sham 884 days, IR 814 days.

**b)** Kaplan-Meier survival curves of sham-treated and irradiated female mice. Irradiation markedly reduced survival in females (P < 0.0001; Gehan-Breslow-Wilcoxon P = 0.0001). Median survival: Sham 875 days, IR 735 days.

**c)** Frailty Index Score (FIS) over time in male mice. Both groups showed significant age-associated increases (Sham slope P = 0.0002; IR slope P < 0.0001).

**d)** Frailty Index Score (FIS) over time in female mice. Both groups demonstrated progressive increases over time (both slopes P < 0.0001).

**e)** Wire hanging test in males showing the percentage of mice successful in the first trial. No difference at 4 months (P = 0.8298), but irradiated males performed worse at 10 months (P = 0.0200).

**f)** Wire hanging test in females. No significant differences between Sham and irradiated females at 4 months (P = 0.3492) or 10 months (P = 0.3351).

**g)** RotaRod performance (speed at fall, rpm) in males at 4 and 10 months after irradiation. No statistically significant difference at 4 months (P = 0.9645), but irradiated males showed reduced performance at 10 months (P = 0.0193).

**h)** RotaRod performance in females. No statistically significant difference between groups at 4 months (P = 0.9964) or 10 months (P = 0.1179).

**i)** Y-maze novel arm exploration in males shown as time spent in the novel arm. Irradiated males (10 months after IR) spent significantly less time in the novel arm compared to sham (P = 0.0277).

**j)** Y-maze novel arm exploration in females. No significant difference between sham and irradiated females (P = 0.1617) at 10 months after IR.

**k)** Latency to enter the novel arm in males. No significant difference between groups (P = 0.1670).

**l)** Latency to enter the novel arm in females. No significant difference between groups (P = 0.2615).

**m)** Stone T-maze performance in males shown as frequency of errors across trial blocks (trials 1-3, 4-6, 7-9). Irradiated males (10 months after IR) exhibited increased errors during trials 1-3 (P = 0.0298), with no statistically significant differences in later blocks (4-6, P = 0.1756; 7-9, P = 0.9744).

**n)** Stone T-maze performance in females. Irradiated females (10 months after IR) showed increased errors during trials 1-3 (P = 0.0007), with no statistically significant differences in later blocks (4-6, P = 0.9109; 7-9, P = 0.8880).

**o)** Time to complete the Stone T-maze in males. Irradiated males required more time during trials 1-3 (P = 0.0020), with no statistically significant differences during trials 4-6 (P = 0.5403) or 7-9 (P = 0.9834).

**p)** Time to complete the Stone T-maze in females. No significant differences between groups across trial blocks (trials 1-3, P = 0.2825; 4-6, P = 0.6000; 7-9, P = 0.9751).

**q)** Time to complete the Stone T-maze in pooled male and female mice. Irradiated mice required more time during trials 1-3 (P = 0.0046), with no statistically significant differences in later blocks (4-6, P = 0.9302; 7-9, P = 0.9998).

Statistical analyses: a, b) Log-rank (Mantel–Cox) test with Gehan-Breslow-Wilcoxon test. c, d) Simple linear regression. e, f) Fisher’s exact test (two-sided). g, h) Mixed-effects model (REML). i-l) Unpaired t-test (Welch’s correction where appropriate). M-q) Ordinary two-way ANOVA with trial block and treatment as factors, followed by Šídák’s multiple comparisons test. Data are presented as mean ± SEM.

Sample sizes: a) Sham n = 16, IR n = 18; b) Sham n = 21, IR n = 20; c) Sham n = 13, IR n = 15; d) Sham n = 21, IR n = 18; e) 4m: Sham n = 44, IR n = 44, 10m: Sham = 13, IR = 15; f) 4m: Sham n = 33, IR n = 29, 10m: Sham n = 19, IR n = 17; g) 4m: Sham n = 16, IR n = 15, 10m: Sham n = 15, IR n = 15; h) 4m: Sham n = 17, IR n = 14, 10m: Sham n = 17, IR n = 13; i) Sham n = 11, IR n = 10; j) Sham n = 13, IR n = 11; k) Sham n = 11, IR n = 10; l) Sham n = 13, IR n = 11; m, n) Sham n = 11, IR n = 11; o) Sham n = 22, IR n = 22; p-q) Sham n = 11, IR n = 11.

Data are presented as mean ± SEM. *P < 0.05, ***P < 0.001, ****P < 0.0001.

**Figure S2. Early senolytic intervention does not significantly prevent functional decline following irradiation.**

**a)** Frailty Index Scores in male mice at 7, 11, 14, and 17 months of age. No statistically significant differences were observed between groups at 7 or 11 months. At 14 months, both IR (P = 0.0227) and IR-AP (P = 0.0006) mice exhibited increased frailty compared to sham. At 17 months, IR (P = 0.0037) and IR-AP (P < 0.0001) mice showed further increases compared to Sham. No significant differences were detected between IR and IR-AP at any time point.

**b)** Frailty Index Scores in female mice at 7, 11, 14, and 17 months of age. No statistically significant differences were observed at 7 or 11 months. At 14 months, both IR (P = 0.0004) and IR-AP (P < 0.0001) mice exhibited increased frailty compared to sham. At 17 months, both IR and IR-AP groups remained significantly elevated compared to sham (both P < 0.0001). No statistically significant differences were detected between IR and IR-AP groups.

**c)** RotaRod performance (speed at fall, rpm) in male mice at 11 and 17 months. No significant differences were detected between sham, IR, and IR-AP groups at either time point.

**d)** RotaRod performance in female mice at 11 and 17 months. No significant differences were observed between groups.

**e)** Time-based Y-maze discrimination index in male mice. Irradiated males 11 months after IR) showed reduced discrimination compared to sham (P = 0.0422), while early AP treatment did not significantly improve performance (P = 0.3042 *vs*. IR).

**f)** Time-based Y-maze discrimination index in female mice. No significant differences were detected between sham, IR, and IR-AP groups at 10 months after IR.

Statistical analyses: a, b) Two-way ANOVA with repeated measures followed by Dunnett’s or Tukey’s multiple comparisons tests as appropriate. c, d) Two-way ANOVA with Tukey’s multiple comparisons test. e, f) One-way ANOVA followed by Dunnett’s multiple comparisons test.

Sample sizes: a) Sham n = 15, IR n = 15, IR-AP n = 16, b) Sham n = 15, IR n = 15, IR-AP n = 16, c) 11m: Sham n = 15, IR n = 15, IR-AP n = 16; 17m: Sham n = 15, IR n = 15, IR-AP n = 16, d) 11m: Sham n = 15, IR n = 15, IR-AP n = 15; 17m: Sham n = 15, IR n = 9, IR-AP n = 9, e) Sham n = 12, IR n = 12, IR-AP n = 12, f) Sham n = 14, IR n = 8, IR-AP n = 9.

Data are presented as mean ± SEM. *P < 0.05, **P < 0.01, ***P < 0.001, ****P < 0.0001.

**Figure S3. Time-dependent neuroinflammatory changes in the hippocampus following whole-body irradiation.**

**a)** Representative images of hippocampal microglia stained for Iba-1 (red) and DAPI (blue) in Sham IR, 1 month post-irradiation (1m IR), and 4 months post-irradiation (4m IR).

**b)** Quantification of microglial numbers in the hippocampus. Microglial number was increased at 1 month compared to Sham (P = 0.0255), with no statistically significant difference between Sham and 4 months (P = 0.8364) or between 1 and 4 months after IR (P = 0.0873).

**c)** IL-2 expression (fold change). Increased at 1 month compared to Sham (P < 0.0001), with no significant difference at 4 months after IR (P = 0.1529 *vs*. Sham).

**d)** IP-10 expression (fold change). Increased at 1 month (P < 0.0001) and remained elevated at 4 months after IR compared to Sham (P = 0.0011).

**e)** M-CSF expression (fold change). No statistically significant change at 1 month (P = 0.5729) but increased at 4 months after IR compared to Sham (P = 0.0097).

**f)** IFN-α expression (fold change). Increased at 1 month compared to Sham (P = 0.0083), with no significant difference at 4 months after IR (P = 0.1677).

**g)** MIP-1 expression (fold change). Increased at 1 month (P = 0.0007), with a trend but no significant difference at 4 months after IR (P = 0.0533).

**h)** MIP-2 expression (fold change). Increased at 1 month (P = 0.0063), with no statistically significant difference at 4 months after IR (P = 0.9546).

**i)** IL-1α expression (fold change). Increased at 1 month (P = 0.0158), with no significant difference at 4 months after IR (P = 0.5002) in comparison to Sham.

**j)** IL-1β expression (fold change). Increased at 1 month (P = 0.0003), with no significant difference at 4 months after IR (P = 0.1529).

**k)** RANTES expression (fold change). Strongly increased at both 1 month and 4 months after IR compared to Sham (both P < 0.0001).

**l)** IL-13 expression (fold change). No statistically significant change at 1 month (P = 0.1193) but increased at 4 months after IR compared to Sham (P = 0.0013).

Open circles represent females and closed circles represent males.

Scale bars = 50μm.

Statistical analyses: b) One-way ANOVA with Tukey’s multiple comparisons test. c-l) One-way ANOVA followed by Dunnett’s multiple comparisons test *vs*. sham.

Data are presented as mean ± SEM (n = 8-10 animals per group). *P < 0.05, **P < 0.01, ***P < 0.001, ****P < 0.0001. .

**Figure S4. Additional characterization of irradiation-induced hepatic damage, inflammation, lipid accumulation, and skeletal muscle pathology.**

**a)** Low-magnification images of liver sections stained for p16 (red) and DAPI (blue) in sham, 1 month, and 4 months after irradiation.

**b)** Low-magnification images of liver sections stained for p21 (red) and DAPI (blue) in sham, 1 month, and 4 months after irradiation.

**c)** Low-magnification images of liver sections stained for F4/80 (red) and DAPI (blue) in sham, 1 month, and 4 months after irradiation.

These images correspond to the higher-magnification images shown in Figure 4 and illustrate the overall distribution of senescent cell and inflammatory markers across the liver tissue. Scale bars: 50μm.

**d)** Representative images of telomere-associated DNA damage foci (TAF) in hepatocytes stained for telomeres (red), γH2A.X (green), and DAPI (blue) in Sham, 1 month, and 4 months after irradiation. Higher magnification images highlight TAF, defined by the colocalization of telomere and γH2A.X signals (yellow). White arrows indicate TAF.

**e)** Quantification of TAF-positive hepatocytes. TAF-positive cells were significantly increased at 1 month (P < 0.0001) and 4 months after IR (P = 0.0315) compared to Sham and were higher at 1 month compared to 4 months after IR (P = 0.0001).

**f)** Quantification of average γH2A.X foci per hepatocyte. Foci number was significantly increased 1 month after IR (P=0.0013) and decreased at 4 months after IR (P=0.0144).

**g)** Quantification of percentage γH2A.X positive hepatocytes. The percentage of γH2A.X positive hepatocytes was significantly increased 1 month after IR (P=0.0025).

**h)** Quantification of p21 and F4/80 double positive cells. Double positive cells were significantly increased 1 month after IR (P=0.0056) and decreased at 4 months after IR (P=0.0082).

**i)** Quantification of muscle triglyceride showed a significant increase at 4-month post-IR (P=0.0472)

**j)** Quantification of centrally nucleated skeletal muscle fibers in Sham, 1-month, and 4-month post-IR mice (P=0.0136).

Scale bars: 50 μm in a-c; 5 μm in d.

Open circles represent females and closed circles represent males.

Statistical analyses: ordinary one-way ANOVA for all panels. Sample sizes: Sham n = 11; 1 month IR n = 10; 4 months IR n =10 animals per group.

Data are presented as mean ± SEM. *P < 0.05, **P < 0.01, ***P < 0.001, ****P < 0.0001.

**Figure S5. Sex-stratified functional and cognitive outcomes following delayed (4m) AP treatment after irradiation.**

**a)** Kaplan-Meier survival curves in male mice. No significant differences were detected between Sham, IR, and IR-AP groups (P = 0.1055). Median survival: Sham 884 days, IR 823 days and IR-AP 830 days.

**b)** Kaplan-Meier survival curves in female mice. Survival differed significantly between groups (log-rank, P < 0.0001). Delayed AP treatment improved survival compared with IR (log-rank, P = 0.0473), while Sham mice had higher survival than IR (P < 0.0001). Median survival: Sham 865 days, IR 696.5 days and IR-AP 743 days.

**c)** Bodyweight, **d)** muscle weight, **e)** liver weight and **f)** brain weight in male and female mice at the end of study.

**g)** Frailty Index Scores in male mice at 10, 14, 17, and 20 months of age. Frailty increased in IR mice relative to Sham at multiple ages (10m P = 0.0010; 14m P < 0.0001; 17m P < 0.0001; 20m P = 0.0020). IR-AP mice were also increased relative to Sham (10m P < 0.0001; 14m P = 0.0121; 17m P = 0.0127; 20m P = 0.0108). No significant differences were detected between IR and IR-AP at any age (all P > 0.05).

**h)** Frailty Index Scores in female mice at 10, 14, 17, and 20 months of age. IR mice showed increased frailty compared with Sham at 10m (P < 0.0001), 14m (P < 0.0001), and 17m (P = 0.0001), while IR-AP mice were also elevated *vs*. Sham at 10m (P < 0.0001), 14m (P = 0.0013), 17m (P < 0.0001), and 20m (P = 0.0461). IR-AP was reduced compared with IR at 14m (P = 0.0014), but no statistically significant differences between IR and IR-AP were detected at 10m, 17m, or 20m (all P > 0.05).

**i)** Individual components contributing to the frailty index, shown for both sexes combined. Sham n=9, IR n=9, IR-AP n=10.

**j)** RotaRod performance (speed at fall, rpm) in male mice. IR mice exhibited reduced performance compared with Sham (P = 0.0304) and IR-AP (P = 0.0230).

**k)** RotaRod performance in female mice. IR-AP mice showed improved performance compared with IR (P = 0.0062), while Sham *vs*. IR was not significant (P = 0.1449).

**l)** Time-based Y-maze discrimination index in male mice. No significant differences were detected between groups (P = 0.6584).

**m)** Time-based Y-maze discrimination index in female mice. IR mice had reduced discrimination compared with Sham (P = 0.0072) and IR-AP (P = 0.0025).

Statistical analyses: a, b) Log-rank (Mantel-Cox) test with Gehan-Breslow-Wilcoxon test. c, d) Ordinary two-way ANOVA with age and treatment as factors, followed by Tukey’s multiple comparisons test. e-h) One-way ANOVA followed by Dunnett’s multiple comparisons test.

Sample sizes: a) Sham n = 16, IR-VH n = 17, IR-AP n = 17 (males). b) Sham n = 21, IR-VH n =20, IR-AP n = 19 (females). g) 10m: Sham n = 13, IR n = 15, IR-AP n = 17; 14m: Sham n = 13, IR n = 15, IR-AP n = 15; 17m: Sham n = 12, IR n = 13, IR-AP n = 13; 20m: Sham n = 11, IR n = 13, IR-AP n = 13 (males). h) 10m: Sham n = 20, IR n = 18, IR-AP n = 16; 14m: Sham n = 20, IR n = 14, IR-AP n = 13; 17m: Sham n = 17, IR n = 13, IR-AP n = 11; 20m: Sham n = 14, IR n = 6, IR-AP n = 6 (females). j) Sham n = 15, IR n = 15, IR-AP n = 15 (males). k) Sham n = 17, IR n = 12, IR-AP n = 11 (females). l) Sham n = 20, IR n = 18, IR-AP n = 21 (males). m) Sham n = 22, IR n = 20, IR-AP n = 17 (females).

Data are presented as mean ± SEM. *P < 0.05, **P < 0.01, ***P < 0.001, ****P < 0.0001.

**Figure S6. Delayed p16^Ink4a^ clearance attenuates irradiation-induced tissue pathology**

**a)** Representative images of p21^Cip1^ with RNA-ISH (red) with DAPI (blue) in Sham, IR, and IR-AP mice.

**b)** Percentage of IL-6^+^ neurons. IR-AP mice showed reduced IL-6^+^ neurons compared to IR (Welch’s t-test, P = 0.0243).

**c)** Percentage of IL-1α^+^ neurons. No significant differences were detected between IR and IR-AP groups (Welch’s t-test, P = 0.7200).

**d)** Quantification of telomere-associated DNA damage foci (TAF^+^) in neurons. Irradiation increased TAF^+^ neurons compared to Sham (P < 0.0001), and AP treatment reduced TAF burden (P = 0.0011 *vs*. IR).

**e)** Representative images of cortical astrocytes stained for p21^Cip1^ with RNA-ISH (red), GFAP (magenta), Glut-1 (green), and DAPI (blue) in Sham-IR, IR, and IR-AP mice.

**f)** Quantification of p16^Ink4a+^ astrocytes. IR mice exhibited increased p16^Ink4a+^ astrocytes compared to Sham (P = 0.0005) and IR-AP (P = 0.0318).

**g)** Quantification of p21^Cip1+^ astrocytes. IR mice showed increased p21^Cip1+^ astrocytes compared to Sham (P = 0.0030), while no significant difference was detected between IR and IR-AP (P = 0.1311).

**h)** Hepatic triglyceride (TG) content in AP treated and control animals (P=0.0012).

**i)** Serum alanine aminotransferase (ALT) in AP treated and control animals (P<0.0001).

**j)** Aspartate aminotransferase (AST) in AP treated and control animals (P=0.0043).

**k)** Quantification of p16^Ink4a+^ hepatocytes. p16^Ink4a^ positivity was significantly decreased after AP treatment (P = 0.0065).

**l)** Quantification of the percentage of γH2A.X positive hepatocytes. Percentage of γH2A.X positive hepatocytes was significantly reduced after AP treatment (P = 0.0007).

**m)** Quantification of the percentage of TAF positive hepatocytes. Percentage of TAF positive hepatocytes was significantly reduced after AP treatment (P = 0.01).

**n)** Quantification of p21^Cip1+^ hepatocytes. p21^Cip1^ was not changed after AP treatment (P = 0.9356).

**o)** Muscle triglyceride (TG) content in AP treated and control animals was not changed (P=0.6773).

**p)** Quantification of p16^Ink4a+^ muscle fibers. p16^Ink4a^ positivity was not changed after AP treatment (P = 0.9418).

**q)** Quantification of p21^Cip1+^ fibers. p21-positive fibers were significantly decreased after AP treatment (P = 0.0360).

Open circles represent females and closed circles represent males. Scale bars: 20μm.

Statistical analyses: d-g) One-way ANOVA followed by Dunnett’s multiple comparisons test. b, c, h-q) Unpaired t-test with Welch’s correction (two-tailed).

Data are presented as mean ± SEM. *P < 0.05, **P < 0.01, ***P < 0.001, ****P < 0.0001.
